# Phenylacetic Acid, a Gut Microbially Produced Metabolite, Reduces Atherosclerosis Burden and Impacts Host Lipid Homeostasis

**DOI:** 10.1161/JAHA.125.047044

**Published:** 2026-07-10

**Authors:** Kelley M. Carr, Jiyeon Kim, Junchul Shin, Kathryn A. Bacik, Junyoung Hong, Irene Krukovets, Christopher M. Strauch, Naseer Sangwan, Olga A. Cherepanova, Ina Nemet

**Affiliations:** ^1^ Department of Cardiovascular and Metabolic Sciences Cleveland Clinic Research Cleveland OH USA; ^2^ Center for Microbiome & Human Health Cleveland Clinic Cleveland OH USA; ^3^ Shared Laboratory Resources Cleveland Clinic Research, Cleveland Clinic Cleveland OH USA; ^4^ Cleveland Clinic Lerner College of Medicine, Case Western Reserve University School of Medicine Cleveland OH USA

**Keywords:** atherosclerosis, cardiovascular disease, gut microbes, phenylacetic acid, phenylacetylglutamine, phenylacetylglycine, Basic Science Research, Biomarkers, Lipids and Cholesterol, Metabolism, Vascular Biology

## Abstract

**Background:**

Gut microbial metabolism of dietary phenylalanine produces phenylacetic acid (PAA), followed by the host conversion to phenylacetylglutamine in humans and phenylacetylglycine in mice. Phenylacetylglutamine was linked to cardiovascular disease risk in multiple clinical studies, yet whether the microbial pathways leading to PAA/phenylacetylglutamine/phenylacetylglycine formation influence atherosclerosis progression within the host remains elusive.

**Methods:**

Atheroprone *Apoe* ^−/−^ mice on a Western diet were provided with a gut microbial metabolite PAA to investigate its effects on host cardiometabolic health.

**Results:**

Circulating levels of phenylacetylglutamine and phenylacetylglycine were increased by the treatment without affecting circulating cholesterol or inflammatory cytokines. In male mice, PAA elevated triglycerides and fasting glucose. PAA decreased the total atherosclerotic plaque burden within the descending and abdominal aortas of both sexes and within brachiocephalic arteries in male mice without affecting plaque stability indices. PAA altered gut microbial composition but did not markedly shift production of established atherosclerosis‐related microbial metabolites, aside from a modest rise in indoxyl sulfate in female mice. Furthermore, PAA treatment decreased circulating levels of acyl‐ and free carnitines through reduced availability of their biosynthetic precursors and upregulated expression of genes involved in peroxisomal lipid metabolism.

**Conclusions:**

These results offer new insights into the impact of gut‐microbial metabolism of phenylalanine on host metabolism and atherosclerosis progression. Our findings suggest that clinical associations between phenylacetylglutamine and cardiovascular disease risk are unlikely to be driven by increased atherosclerosis.

**Nonstandard Abbreviations and Acronyms**
4HPA: 4‐hydroxyphenylacetic acid
ACTA2: smooth muscle α‐actin 2
ATF3: activating transcription factor 3
CD31: platelet endothelial cell adhesion molecule‐1
GBB: γ‐butyrobetaine
LC–MS/MS: liquid chromatography tandem mass spectrometry
LGALS3: galectin‐3
MRM: multiple reaction monitoring
PAA: phenylacetic acid
PA‐Car: phenylacetyl‐carnitine
SCFA: short‐chain fatty acid
TMAO: trimethylamine *N*‐oxide
WT: wild‐type

**Research Perspective**

**What Is New?**

- Dietary provision of phenylacetic acid to atheroprone mice reduces atherosclerotic burden.
- Phenylacetic acid treatment decreased circulating levels of esterified and free carnitines.

**What Question Should Be Addressed Next?**

- Because phenylacetic acid reduces the total carnitine pool, testing whether restoring carnitine levels will abolish phenylacetic acid effects on atherosclerosis progression.

Cardiovascular disease (CVD) is the leading cause of death and disability worldwide despite advancements in cholesterol‐lowering treatments.^1^ Elucidating pathways that contribute to CVD, beyond traditional risk factors, is therefore essential for developing more effective strategies to prevent and reduce its progression. Multiple diseases, including CVD, obesity, type 2 diabetes, metabolic syndrome, and hypertension are associated with altered gut microbiome structure and function.^2^ Microbial fermentation of dietary fibers into short‐chain fatty acids (SCFAs) has been associated with improved host metabolism.^3^ Moreover, when provided as a supplement, SCFA propionate not only reduced cholesterol levels in animal models but also in humans enrolled in a randomized, double‐blinded, placebo‐controlled trial.^4^ In contrast, trimethylamine *N*‐oxide (TMAO), a gut microbial metabolite produced from nutrients enriched in red meat and Western diets, contributed to atherosclerosis and thrombosis in mouse models and has been associated with CVD in humans.^5^ Although the microbial production of SCFAs and TMAO can, to some extent, be modified by respective increases in fiber intake or decreases in red meat consumption, gut microbes also produce derivatives from essential nutrients.

We have recently shown that a gut microbial metabolite derived from the essential amino acid phenylalanine, phenylacetylglutamine, is associated with incident risk for major adverse cardiovascular events (defined as myocardial infarction, stroke, or death).^6^ Additional follow‐up studies further demonstrated clinical associations between phenylacetylglutamine and heart failure,^7^, ^8^ coronary atherosclerotic burden among individuals with suspected coronary artery disease (CAD),^9^ in‐stent stenosis and hyperplasia in subjects with CAD,^10^ and incident coronary heart disease risk among women.^11^ Phenylacetylglutamine levels were also higher in patients with an unexplained residual score (a high plaque burden not explained by the traditional risk factors) when compared with patients with a protected residual score (less plaque than predicted by the traditional risk factors).^12^ Although these correlative and associative studies provide growing evidence that microbial metabolism of phenylalanine may contribute to CVD development and progression, a causal link between the gut microbial pathways that lead to phenylacetylglutamine production and host cardiometabolic health remains elusive.

Phenylalanine, as an essential amino acid, is important not only for maintaining the structural and functional integrity of cells as a protein building block but also as a direct precursor of tyrosine, monoamine neurotransmitters such as dopamine, norepinephrine (noradrenaline), and epinephrine (adrenaline), and the natural pigment melanin. Dietary phenylalanine that evades host absorption is metabolized by microbes residing within the digestive tract. Microbes first deaminate phenylalanine into phenylpyruvic acid followed by its oxidation into phenylacetic acid (PAA).^13^ Phenylacetylglutamine and phenylacetylglycine are products of respective conjugation of glutamine, the predominant pathway in humans, and glycine, the main pathway in mice, to the microbially produced PAA by the host hepatorenal enzymes^6^, ^14^ (Figure 1A). Besides being elevated in individuals with CVD, PAA and its downstream metabolites are elevated in older people,^15^, ^16^ nondiabetic obese women,^17^ people with end‐stage renal disease,^18^ and individuals with phenylketonuria,^19^ a rare congenital error in phenylalanine metabolism.

**Figure 1:**
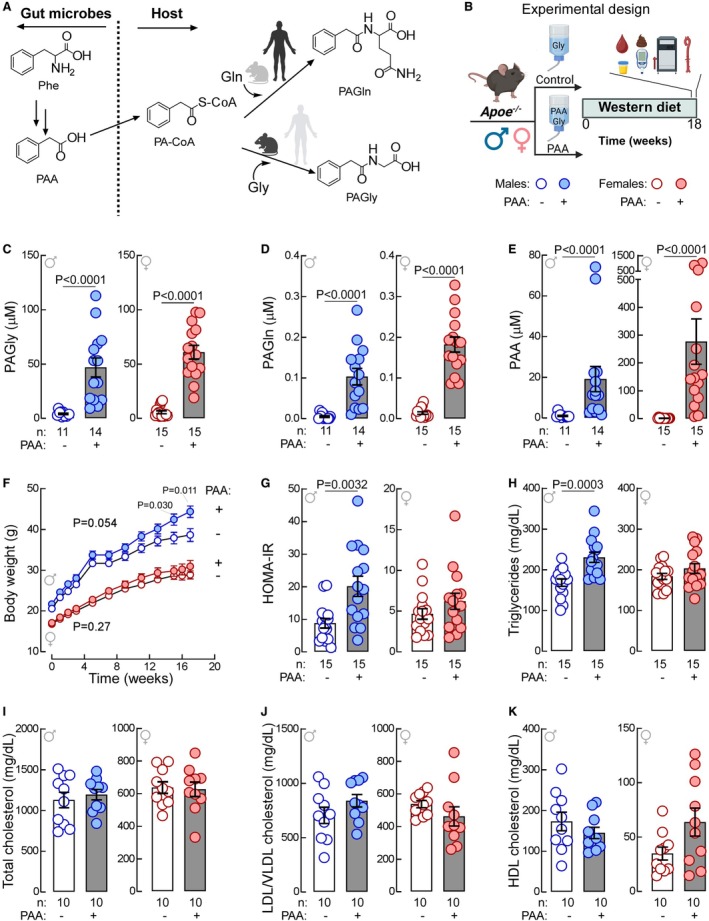
Dietary provision of PAA impairs systemic metabolism of *Apoe* ^−/−^ male mice on a Western diet. **A**, Metaorganismal production of PAA by gut microbes with subsequent conjugation to amino acids Gln and Gly by the host liver enzymes and respective production of PAGln and PAGly. **B**, Experimental design. **C** through **E**, Circulating levels of PAGly, PAGln, and PAA in male (blue) and female (red) *Apoe* ^−/−^ mice on a Western diet in the fed state with (● symbols) and without (○ symbols) dietary provision of PAA. **F**, Body weight over time in male (blue) and female (red) *Apoe* ^−/−^ mice on a Western diet with (● symbols) and without (○ symbols) dietary provision of PAA. **G** through **K**, HOMA‐IR and plasma lipid profiles in male (blue) and female (red) *Apoe* ^−/−^ mice on a Western diet with (● symbols) and without (○ symbols) dietary provision of PAA. Data were analyzed by Mann‐Whitney test (**C** through **E**, **G** through **K**) or mixed‐model ANOVA with Tukey post hoc test (**F**). Values are mean±SEM; n=10–15, as indicated. See also Figure S1. Gln indicates glutamine; Gly, glycine; HDL, high‐density lipoprotein; HOMA‐IR, homeostatic model assessment of insulin resistance; LDL, low‐density lipoprotein; PAA, phenylacetic acid; PA‐CoA, phenylacetyl‐coenzyme A; PAGln, phenylacetylglutamine; PAGly, phenylacetylglycine; Phe, phenylalanine; and VLDL, very low‐density lipoprotein.

Although the correlative and associative clinical studies provide growing evidence that microbial metabolism of phenylalanine may contribute to the development and progression of CVD and its comorbidities, a causal link between gut microbial pathways that lead to phenylacetylglutamine/phenylacetylglycine production and host cardiometabolic health remains largely unexplored. Moreover, the only *in vivo* mechanistic studies in animal models on the role of phenylacetylglutamine/phenylacetylglycine in the context of CVD have involved acute, intraperitoneal injections of phenylacetylglutamine or phenylacetylglycine, which lead to increased platelet responsiveness^6^ or induced expression of the B‐type natriuretic peptide gene in the left mouse atria.^7^ Additionally, phenylacetylglutamine intraperitoneal injections twice daily for 20 days caused a significant elevation in left ventricular end‐diastolic filling pressure and reduction in plasma and tissue nitrite in the phenylacetylglutamine‐treated mouse cohort.^20^ However, the effects of chronic exposure to PAA, the direct microbial product and precursor of phenylacetylglutamine/phenylacetylglycine, on host cardiovascular health have not been studied. Phenylacetylglutamine and phenylacetylglycine are not only cleared from circulation in <30 minutes when delivered intraperitoneally,^6^ but they bypass normal host metabolic processing.

Here, we show that dietary provision of PAA (bypassing gut microbes) decreased atherosclerotic plaque burden in apolipoprotein E knockout (*Apoe* ^−/−^) mice without affecting indices of plaque stability. Although PAA supplementation affected gut microbial composition, microbial community output of metabolites associated with CVD was not markedly altered. Instead, PAA supplementation caused an imbalance in carnitine homeostasis, a phenotype also observed in the nonatherosclerotic (C57BL/6J) wild‐type (WT) animals, and upregulated expression of genes encoding peroxisomal fatty acid oxidation enzymes.

These results provide new insights into the gut‐microbial metabolism of phenylalanine and its impact on host cardiometabolic physiology. Despite strong associations between phenylacetylglutamine and CVD risk in clinical cohorts, our findings suggest that clinical associations between phenylacetylglutamine and CVD risk are unlikely to be driven by increased atherosclerotic burden.

## METHODS

### Data Availability Statement

Raw sequencing data for 16S ribosomal RNA generated in this study are available on Zenodo (DOI: 10.5281/zenodo.15632604). Any additional information required to reanalyze the data reported in this article is available from the corresponding author upon request.

### Ethics Statement

All animal model studies were approved by the Institutional Animal Care and Use Committee at the Cleveland Clinic.

### Mice

Male and female *Apoe* ^−/−^ (n=15 per group) and C57BL/6J (WT) (n=10 per group) mice were obtained from Jackson Laboratories and housed in standard 12‐hour light/dark housing within the Cleveland Clinic Lerner Research Building mouse facility. Mice were randomly housed with 5 mice per cage upon arrival. The mice were randomized into cages by personnel blinded to the study experimental design. *Apoe* ^−/−^ mice were fed a Western diet (Teklad Custom Research Diets, Madison, WI; catalog number TD.88137) consisting of 0.2% cholesterol, 17.3% protein, 48.5% carbohydrate, and 21.2% fat (42.0% kcal from fat). WT mice were fed a high‐fat diet (Research Diets, New Brunswick, NJ; catalog number D12492i) consisting of 31.7% fat (60.0% kcal from fat). PAA (1% in water, w/w; Sigma‐Aldrich, St. Louis, MO; catalog number W287806) neutralized with sodium bicarbonate (Sigma‐Aldrich; catalog number S6297) and supplemented with an equimolar amount of glycine (Sigma‐Aldrich, catalog number G7126) was administered through drinking water. With this dose of PAA, circulating levels of phenylacetylglycine reached values equivalent to phenylacetylglutamine observed in clinical studies.^6^, ^21^, ^22^, ^23^ The control group received an equivalent amount of sodium glycinate (Sigma‐Aldrich; catalog number G6761). Mouse body weight was measured weekly in C57BL/6J and biweekly in *Apoe* ^−/−^ mice. In WT mice, food intake was assessed by weighing the food consumed weekly, divided by the number of mice in each cage. Personnel were not blinded to the treatment groups. Additional methodological details for the animal experiments are provided in Data S1.

Nonfasting blood was collected between 7:00 am and 9:00 am in heparin‐coated capillaries (Fisher Scientific, Pittsburgh, PA; catalog number 22‐362‐566) from the saphenous vein. Blood was centrifuged (1500*g*, 10 minutes, 4 °C), and clear plasma supernatant was aliquoted and stored at −80 °C before analysis. Body composition (expressed as percent of fat mass and percent of lean mass) was assessed at baseline and at the conclusion of the study using EchoMRI body composition analysis (EchoMRI, Houston, TX).

*Apoe* ^−/−^ mice were fasted overnight and euthanized via CO_2_ asphyxiation, followed by cervical dislocation and blood collection via cardiac puncture. The brachiocephalic artery and aorta dissected from the aortic arch to the iliac bifurcation were harvested and fixed in 4% paraformaldehyde (Electron Microscopy Sciences, Hatfield, PA; catalog number 15710). Brachiocephalic arteries were embedded in paraffin and sectioned at 5 μm thickness. Morphometric and immunohistochemical analyses were performed using 1 to 4 sections per artery. Descending thoracic and abdominal aortas were subjected to Sudan IV staining. WT mice were euthanized in the fed state between 7:00 am and 10:00 am by anesthesia overdose (>300 mg/kg ketamine+30 mg/kg xylazine), blood was collected by cardiac puncture, and tissues were harvested, flash‐frozen, and stored at −80 °C until the time of analysis.

### Measuring Fasting Glucose, Insulin, Homeostatic Model Assessment of Insulin Resistance, Circulating Lipid Profile, and Circulating Cytokines

Glucose was measured using a handheld Accu‐Chek Guide blood glucose monitor (model 897; Roche Diagnostics Operations, Indianapolis, IN) via tail tip cut before euthanasia. Insulin was measured by ultrasensitive mouse insulin ELISA kit (Crystal Chem, Elk Grove Village, IL; catalog number 90080) following manufacturer instructions. Homeostatic model assessment for insulin resistance was calculated as: (insulin [μIU/mL] × blood glucose [mmol/L])/22.5. Total plasma triglyceride levels were measured on the Roche Cobas 6000 automated chemistry analyzer (Roche Diagnostics Operations). Total high‐density lipoprotein (HDL) and low‐density lipoprotein (LDL)/very low‐density lipoprotein (VLDL) plasma cholesterol were measured by HDL and LDL/VLDL colorimetric assay kit (LifeSpan BioSciences, Shirley, MA; catalog number LS‐K314‐100) following manufacturer instructions. Circulating cytokines were measured by multiplexing V‐PLEX plus proinflammatory panel 1 mouse kit (MesoScale Discovery, Rockville, MD; catalog number K15048G‐1).

### Lipid Accumulation Analysis by Sudan IV Staining

Aortas from *Apoe* ^−/−^ mice dissected from the aortic arch to the iliac bifurcation were subjected to *en face* Sudan IV staining. After meticulously cleaning the periadventitial fat, aortas were dehydrated in 70% ethanol for 5 minutes and incubated in Sudan IV solution for 6 minutes, which was prepared as follows: 1 g Sudan IV (Sigma‐Aldrich; catalog number S4261) was diluted in 100 mL 70% ethanol and 100 mL 100% acetone (Alfa Aesar, Ward Hill, MA; catalog number AA22928K7). Finally, aortas were differentiated in 80% ethanol for 3 minutes and stored in phosphate‐buffered saline at 4 °C protected from light. One aorta from the female control group was identified as a statistical outlier (77.2% Sudan IV‐positive lesion area) and 1 aorta was damaged during the isolation.

### Morphometric and Immunohistochemical Analyses

Masson Trichrome staining was performed for morphometric analysis. Slides were deparaffinized in Clear Rite 3 (Epredia, Kalamazoo, MI; catalog number 6905), washed twice in 100% ethanol (Fisher Scientific, Hampton, NH; catalog number BP2818‐4), followed by 2 washes in 95% ethanol in distilled water at rt. Slides were then incubated in Bouin's fluid (1 hour, 56 °C; Electron Microscopy Sciences; catalog number 26367‐01) followed by working Weigert's iron hematoxylin (10 minutes, rt; Electron Microscopy Sciences, catalog number 26044‐06 [for A] and 26044‐05 [for B]) and in Biebrich Scarlet acid fuchsin (5 minutes, rt; Electron Microscopy Sciences; catalog number 26033‐02). After each step, slides were washed under a gentle stream of distilled water for 3, 5, and 0.5 minutes, respectively. For the final stain, slides were submerged in phosphotungstic/phosphomolybdic acid (5 minutes, rt; Electron Microscopy Sciences; catalog number 26367‐05), followed immediately with submersion in Aniline Blue (5 minutes, rt; Electron Microscopy Sciences; catalog number 26027‐20) and rinsing in glacial acetic acid in distilled water (1%, 1 minute) and distilled water for 0.5 minutes. Finally, slides were rinsed with 100% ethanol (2×, 1 minute) and xylene (3×, 1 minute; Epredia; catalog number 6601). Dry slides were cover slipped with Cyto‐Seal XYL (Epredia; catalog number 8312‐4), allowed to dry at rt overnight, and imaged by an IX83 Olympus microscope with an Olympus DP80 Camera. The areas within the external elastic lamina, internal elastic lamina, lesion, and lumen were measured.

Collagen content was observed by PicroSirius Red staining. Briefly, slides were deparaffinized in Clear Rite 3 (Epredia; catalog number 6905), washed 2 times in 100% ethanol (Fisher Scientific, Hampton, NH; catalog number BP2818‐4), followed with 2 washes in 95% ethanol in distilled water at rt. Slides were then incubated in working Weigert iron hematoxylin (8 minutes, rt) and PicroSirius Red (1 hour, rt; 0.1% w/v Sirius Red (Direct Red 80, Sigma‐Aldrich; catalog number 36554‐8) in saturated picric acid (Ricca Chemical Company, Arlington, TX; catalog number 5860‐1) and rinsed with glacial acetic acid in distilled water (0.5%, 2×, 1 minute). Excess water was shaken off the slides, which were rinsed in 100% ethanol (3×, 0.5 minutes) and xylene (3×, 1 minute; Epredia; catalog number 6601). Dry slides were cover‐slipped with Cyto‐Seal XYL (Epredia; catalog number 8312‐4), allowed to dry at rt overnight, and imaged using an IX83 microscope with a polarized lens (Olympus, Waltham, MA) with the same exposure time for all slides. The fibrous cap area was defined as the area encompassing the inner 30 μm layer of the fibrous cap overlying lesions.^24^ Samples were excluded from analysis if the lumen line was unclear or if the lesion was dislocated.

Immunohistochemistry was performed with the following antibodies: rat monoclonal anti‐galectin‐3 (LGALS3; Cedarlane, Burlington, Canada; catalog number CL8942AP, 1:500), rabbit polyclonal CD31 (anti‐platelet endothelial cell adhesion molecule‐1; Abcam, Cambridge, United Kingdom; catalog number 124432, 1:500), mouse monoclonal anti‐α‐smooth muscle, ACTA2 (smooth muscle α‐actin 2) Cy3 (Sigma‐Aldrich; catalog number C6198, 1:500), and rabbit monoclonal ATF3 (anti‐activating transcription factor 3; Cell Signaling, Danvers, MA; catalog number 18665, 1:100). Sections were counterstained with DAPI (Invitrogen, Carlsbad, CA; catalog number D21490). The secondary antibodies were donkey anti‐rabbit Alexa Fluor 488 (Invitrogen; catalog number A21206, 1:100, 1:250, and 1:500) and donkey anti‐rat DyLight 650 (Invitrogen; catalog number SA5‐10029, 1:250).

### Image Analysis

Image‐Pro 10 and ImageJ 1.51k were used for image analysis. All images were taken with an Olympus IX83 coupled with CellSens Dimension software and uploaded as tagged image files. Each image was traced using the polygon tool in Image‐Pro, yielding a total of 3 regions of interest. The 3 regions include the external elastic lamina, the internal elastic lamina, and the lesion. Each specimen was calibrated and standardized via a 100 μm scale bar. The total area of each regions of interest was measured. Image analysis was done by personnel who were not blinded to the treatment details before data analysis.

### Liquid Chromatography Tandem Mass Spectrometry and Gas Chromatography Tandem Mass Spectrometry Analyses

All liquid chromatography tandem mass spectrometry (LC–MS/MS) analyses were performed on a chromatographic system consisting of 2 Shimadzu LC‐30 AD pumps (Nexera X2), a CTO 20AC oven operating at 40 °C, and a SIL‐30 AC‐MP autosampler in tandem with an 8050 triple quadrupole mass spectrometer (Shimadzu Scientific Instruments). The following ion source parameters were applied: nebulizing gas flow, 3 L/min; heating gas flow, 10 L/min; interface temperature, 300 °C; desolvation line temperature, 250 °C; heat block temperature, 400 °C; and drying gas flow, 10 L/min. LabSolutions Version 5.123 software (Shimadzu) was used for data analysis. Gas chromatography tandem mass spectrometry analysis was performed on a Trace 1310 gas chromatograph (ThermoFisher Scientific) in tandem with a Thermo TSQ‐Evo triple quadrupole.

#### Gut Microbial Metabolites

Selected gut microbial metabolites were quantified by a previously published stable‐isotope‐dilution LC–MS/MS method^21^ with some modifications. Briefly, stable‐isotope‐dilution LC–MS/MS was used for quantification of metabolites in (20 μL) plasma. An ice‐cold methanolic solution of internal standards (80 μL; D_5_‐phenylacetylglutamine; D_5_‐phenylacetylglycine, D_5_‐PAA; D_6_‐4‐hydroxyphenylacetic acid; D_7_‐*p*‐cresol sulfate; D_4_‐indoxyl sulfate; D_3_‐creatinine, and D_9_‐TMAO) was added to 20 μL of plasma samples, followed by vortexing and centrifuging (21 000*g*; 4 °C for 15 minutes). The clear supernatants were then transferred to glass vials with micro‐inserts. An XSelect HSS T3 column (3.0×100 mm; 3.5 μm; Waters, Ireland; catalog number 186004780) was used for chromatographic separation. A gradient of solvent A (0.1% acetic acid in water) and solvent B (0.1% acetic acid in acetonitrile) was used for chromatographic separation with a flow rate of 0.4 mL/minute and a 1 μL injection volume. Electrospray ionization in positive and negative ion mode with multiple reaction monitoring (MRM) was performed under the following conditions: *m/z* 265.2 → 130.1 for phenylacetylglutamine; *m/z* 271.0 → 130.1 for D_5_‐phenylacetylglutamine; *m/z* 193.8 → 76.1 for phenylacetylglycine; *m/z* 198.9 → 76.1 for D_5_‐phenylacetylglycine; *m/z* 76.0 → 59.1 for TMAO; *m/z* 85.0 → 66.2 for D_9_‐TMAO; *m/z* 113.9 → 44.2 for creatinine and *m/z* 117.0 → 47.2 for D_3_‐creatinine in positive ion mode and *m/z* 135.0 → 91.0 for PAA; *m/z* 140.2 → 95.9 for D_5_‐PAA; *m/z* 212.0 → 79.8 for indoxyl sulfate; *m/z* 216.0 → 79.9 for D_4_‐indoxyl sulfate; *m/z* 187.0 → 106.9 for *p*‐cresol sulfate; *m/z* 193.9 → 114.1 for D_7_‐*p*‐cresol sulfate; *m/z* 150.9 → 106.9 for 4‐hydroxyphenylacetic acid (4HPA), and *m/z* 157.1 → 112.9 for D_6_‐4‐hydroxyphenylacetic acid in negative ion mode.

#### Gas Chromatography Tandem Mass Spectrometry Analysis of SCFAs

SCFA analysis was performed as previously described.^25^ Briefly, fecal contents were weighed and hydrated with a water solution of NaOH (5 mM). After 40 minutes of vortex‐mixing and centrifugation, 50 μL of the supernatants were transferred to gas chromatography vials containing NaOH (5 mM in water) and isotopically labeled internal standards (5 μL; ^13^C_2_‐acetic acid, D_7_‐butyric acid, D_3_‐lactic acid, and D_2_‐propionic acid). A solution of 2‐butanol/pyridine (50 μL; 3:2; v/v) and isobutyl chloroformate (10 μL) was added, the mixture was vortexed and sonicated, the derivatized acids were extracted with hexane (50 μL), and the hexane layer was transferred into another vial for the gas chromatography tandem mass spectrometry analysis. The quantification of acetic acid, butyric acid, lactic acid, and propionic acid were performed using isotope dilution gas chromatography tandem mass spectrometry by using MRM mode. The absolute quantity of each SCFA was determined using calibration curves measured for each analyte. Chromatographic separation was achieved by using an Rtx‐5MS column coated with 5% phenylmethyl siloxane (30 m×0.250 mm×0.25 μm; Restek, Bellefonte, PA; catalog number 12623‐127). Each extract (1 μL) was injected in split mode (10:1). Helium was used as the carrier gas, with a flow of 1 mL/min. The GC oven temperature program was as follows: the initial temperature of 40 °C was held for 2 minutes after injection before it was increased to 50 °C at 3 °C/min, followed by an increase to 110 °C at 5 °C/min, then 250 °C at 30 °C/min and 310 °C at 70 °C/min, and then held at 310 °C for 3 minutes. Argon was used as a collision gas. The injector, transfer line, and ion source temperatures were set at 260 °C, 290 °C, and 230 °C, respectively. The mass spectrometer was tuned to an electron impact ionization energy of 70 eV in the MRM mode with the following parent to daughter ion transitions: *m/z* 61.0 → 43.0 for acetic acid, *m/z* 63.0 → 45.0 for ^13^C_2_‐acetic acid, *m/z* 71.0 → 41.0 for butyric acid, *m/z* 78.1 → 46.1 for D_7_‐butyric acid, *m/z* 135.1→ 45.1 for lactic acid, *m/z* 138.1 → 48.0 for D_3_‐lactic acid, *m/z* 75.1 → 57.0 for propionic acid, and *m/z* 77.1 → 59.0 for D_2_‐propionic acid.

#### Acylcarnitines

A stable‐isotope‐dilution LC–MS/MS method was used for quantification of acylcarnitines in mouse plasma and urine. An ice‐cold methanolic solution (80 μL) of internal standards (D_3_‐L‐carnitine, acetyl‐D_3_‐L‐carnitine, butyril‐D_7_‐L‐carnitine, hexanoyl‐D_3_‐L‐carnitine, and hexadecanoyl‐D_3_‐L‐carnitine) was added to mouse plasma or urine (20 μL), vortexed, and centrifuged (14 000*g*, 15 minutes, 4 °C). Supernatants were transferred to glass vials with micro‐inserts and subjected to LC–MS/MS analysis. Calibration standards were prepared in methanol, and quality control samples were prepared from pooled plasma samples and processed identically to the samples. LC–MS/MS analysis was performed on the same chromatographic system as described above. A Kinetex HILIC column (100×2.1; 2.6 μm; Phenomenex, Torrance, CA; catalog number PRD‐509459) was used for chromatographic separation. A gradient of 10 mM ammonium formate and 0.1% formic acid in water (solvent A) and 10 mM ammonium formate and 0.1% formic acid in 95% acetonitrile (solvent B) was used for chromatographic separation with a flow rate of 0.4 mL/min and 1 μL injection volume. Electrospray ionization in positive ion mode was used with the following MRM conditions: *m/z* 162.0 → 103.1 for L‐carnitine; *m/z* 165.0 → 63.0 for D_3_‐L‐carnitine; *m/z* 204.1 → 85.0 for acetyl‐L‐carnitine; *m/z* 207.0 → 85.1 for acetyl‐D_3_‐L‐carnitine; *m/z* 218.1 → 85.0 for propionyl‐L‐carnitine; *m/z* 232.1 → 85.0 for butyryl‐L‐carnitine and *iso*‐butyryl‐L‐carnitine; *m/z* 238.9 → 85.1 for butyryl‐D_7_‐L‐carnitine; *m/z* 246.1 → 85.0 for pentanoyl‐L‐carnitine and *iso*‐pentanoyl‐L‐carnitine; *m/z* 260.1 → 85.0 for hexanoyl‐L‐carnitine; *m/z* 263.0 → 85.0 for hexanoyl‐D_3_‐L‐carnitine; *m/z* 288.1 → 85.0 for octanoyl‐L‐carnitine; *m/z* 316.2 → 85.0 for decanoyl‐L‐carnitine; *m/z* 344.1 → 85.0 for dodecanoyl‐L‐carnitine; *m/z* 372.2 → 85.0 for tetradecanoyl‐L‐carnitine; *m/z* 400.2 → 85.0 for hexadecanoyl‐L‐carnitine; *m/z* 403.0 → 85 for hexadecanoyl‐D_3_‐L‐carnitine; *m/z* 428.3 → 85.0 for octadecanoyl‐L‐carnitine, and *m/z* 279.9 → 85.0 for phenylacetyl‐carnitine (PA‐Car).

Short‐chain acylcarnitines were calculated by summing the concentrations of individual acylcarnitines containing 2 to 5 carbon atoms in the fatty acid chain (C_2_–C_5_). Medium‐chain acylcarnitines were calculated by summing the concentrations of acylcarnitines containing 6 to 12 carbon atoms in the fatty acid chain (C_6_–C_12_), and long‐chain acylcarnitines were calculated by summing the concentrations of acylcarnitines containing 14 to 18 carbon atoms in the fatty acid chain (C_14_–C_18_). Fractional renal excretion of carnitine was calculated from quantified plasma (P) and urine (U) levels of carnitine and creatinine as follows: fractional renal excretion=(U_carnitine_ × P_creatinine_)/(P_carnitine_ × U_creatinine_) × 100.

#### Trimethyllysine and γ‐Butyrobetaine (GBB)

Trimethyllysine and GBB were measured by a stable‐isotope‐dilution LC–MS/MS method. An ice‐cold methanolic solution (80 μL) of internal standards (D_9_‐trimethyllysine and D_9_‐GBB) was added to liver methanolic extracts (20 μL) then vortexed and centrifuged (14 000*g*, 15 minutes, 4 °C). LC–MS/MS analysis was performed on the same chromatographic system as described above. A Luna Silica (100×3; 3 μm; Phenomenex; catalog number PRD‐594661) was used for chromatographic separation. A gradient of 0.1% acetic acid in water (solvent A) and 0.1% acetic acid in methanol (solvent B) was used for chromatographic separation with a flow rate of 0.4 mL/min and 1 μL injection volume. Electrospray ionization in positive ion mode was used with the following MRM conditions: *m/z* 189.1 → 84.1 for trimethyllysine; *m/z* 198.3 → 84.1 for D_9_‐trimethyllysine; *m/z* 146.0 → 60.1 for GBB, and *m/z* 155.0 → 69.2 for D_9_‐GBB.

### 16S Analysis

The microbial DNA was extracted from fecal samples using the DNeasy PowerSoil ProKit (Qiagen, Germantown, MD; catalog number 47016), following the manufacturer's instructions. The DNA samples were subjected to 16S rDNA gene amplification and sequencing using methods previously described.^26^ Raw 16S amplicon sequence and metadata were demultiplexed using split_libraries_fastq.py script implemented in QIIME1.9.1.^27^ Demultiplexed fastq file was split into sample‐specific fastq files using split_sequence_file_on_sample_ids.py script from Qiime1.9.1.60. Individual fastq files without nonbiological nucleotides were processed using the divisive amplicon denoising algorithm 2 pipeline.^28^

The output of the divisive amplicon denoising algorithm 2 pipeline (feature table of amplicon sequence variants (ASV)) was processed for α and β diversity analysis using phyloseq,^29^ and microbiomeSeq (http://www.github.com/umerijaz/microbiomeSeq) packages in R.

### Gene Expression Analysis

For real‐time quantitative polymerase chain reaction analyses ≈30 mg of liver tissues, either flash frozen or stored in RNAlater (ThermoFisher Scientific, Vilnius, Lithuania; catalog number AM7021), were homogenized in TRIzol (1 mL; Qiagen; catalog number 79306) followed by the addition of chloroform (200 μL; ThermoFisher Scientific, Fair Lawn, NJ; catalog number 268320025) and centrifugation (20 000*g*, 15 minutes, 4 °C). The aqueous layer was carefully removed and added to 100% isopropanol, vortexed for 15 seconds, incubated at −20 °C for at least 1 hour, and then centrifuged (20 000*g*, 15 minutes, 4 °C). The resulting pellet was washed twice in 75% (v/v) ethanol (Greenfield Global, Shelbyville, KY; catalog number 111000200), air dried, and resuspended in nuclease‐free water (Life Technologies, Austin, TX; catalog number R0601).

Concentrations of high‐purity RNA were measured using Nanodrop (ThermoFisher Scientific, ND‐2000). Genomic DNA was removed using the DNA‐free kit (Invitrogen; catalog number AM1906). Reverse transcription to generate cDNA was performed with the High‐Capacity cDNA Reverse Transcription Kit (ThermoFisher Scientific; catalog number 4374966). The resulting cDNA was diluted 20× (≈100 ng/μL), mixed with Radiant SYBR Green Master Mix (Alkali Scientific, Fort Lauderdale, FL; catalog number QS1020), and quantified via real‐time quantitative polymerase chain reaction using an Applied Biosystems Step One Plus thermocycler (ThermoFisher Scientific). The fold changes in mRNA expression were calculated based on the ∆∆‐cycle threshold (CT) method. Primers used for quantitative polymerase chain reaction are listed in Table S1. For *Pparγ*, Taqman primers (ThermoFisher Scientific; 6‐carboxylfluorescein‐minor groove binder (FAM‐MGB), catalog number Mm00440940_m1) relative to 18S (Rn18s; Rn4+; ThermoFisher Scientific; VIC‐probe label, catalog number Mm03928990_g1) were used instead.

### Statistical Analysis

The Mann‐Whitney test was used for 2‐group comparisons due to the small sample size (n≤15).^30^ Two‐way ANOVA with Tukey post hoc test was used to evaluate sex differences.^31^, ^32^ Linear mixed‐model ANOVA with Tukey or Šídák post hoc tests were used for comparing different groups (PAA treatment versus control) over time (body weight analysis) or brachiocephalic artery cross‐sections at different distances from the aortic arch (morphometric analysis). The mixed‐model was chosen due to an unbalanced number of repeats in some test groups. GraphPad Prism version 10.1.2 software was used to perform statistical analyses. A *P* value of <0.05 was predetermined as the threshold for statistical significance. All data are presented as mean±SD or SEM, as indicated. Statistical tests used to compare conditions are also indicated in the figure legends.

For microbiome statistical analysis, differential abundance analysis was performed using methods selected via benchmarking with the DAtest R package,^33^ which avoids circularity by evaluating methods on simulated data rather than the true biological signal. Briefly, group labels were shuffled to remove real differences, artificial differential abundance was spiked into a known subset of features at a defined effect size, and methods were tested across multiple iterations for recovery of spiked features. Performance was ranked using a composite score based on area under the curve, empirical power (after false discovery rate correction), and false discovery rate control among nonspiked features. Based on the DAtest's benchmarking, we selected linear discriminant analysis effect size and ANOVA as the methods of choice to perform differential abundance analysis. Because benchmarking used these independent, shuffled‐and‐spiked simulations (preserving data set compositionality, library sizes, and sparsity), method selection remained decoupled from the actual study signal, preventing data leakage and spurious overprecision. In our analysis, linear discriminant analysis effect size and ANOVA ranked highest and were applied only once to the original, unperturbed data set to identify differentially abundant features.

We assessed the statistical significance (*P*<0.05) throughout, and whenever necessary, we adjusted *P* values for multiple comparisons according to the Benjamini‐Hochberg method to control the false discovery rate.^34^

To assess β diversity (Bray‐Curtis dissimilarity) patterns, we conducted canonical correspondence analysis using the vegan package in R,^35^ ordinating samples to highlight structuring factors. To evaluate group differences in β diversity and variance explained, we applied permutational multivariate analysis of variance via adonis2 in vegan, yielding pseudo‐F, *R* ^2^, and *P* values from 999 permutations (significance at *P*<0.05).

## RESULTS

### Dietary Provision of PAA Reduces Atherosclerosis Burden

To test whether microbial pathways that result in PAA production affect atherosclerosis progression within the host, PAA was provided to atheroprone *Apoe* ^−/−^ male and female mice on a Western diet through drinking water for 18 weeks. Because glycine deficiency enhances atherosclerosis progression in *Apoe* ^−/−^ mice^36^ and because PAA could potentially deplete glycine through conjugation during phenylacetylglycine production by the host (Figure 1A), glycine was also supplied in the drinking water at an equimolar amount to PAA.^6^ Additionally, because PAA is only soluble in water as a salt, an equivalent amount of glycine in the form of its sodium salt (Na‐glycinate) was added to the drinking water of the control group (Figure 1B). In agreement with the rodent metabolic pathways, dietary provision of PAA increased circulating levels of phenylacetylglycine (Figure 1C) and, to a lesser extent, phenylacetylglutamine (Figure 1D), as well as free, unmetabolized PAA (Figure 1E). Notably, phenylacetylglycine levels were equivalent to phenylacetylglutamine levels observed in clinical studies.^6^, ^21^, ^22^, ^23^ Although phenylacetylglycine reached similar plasma levels in both sexes, phenylacetylglutamine levels were higher in female than male mice (*P*<0.001, 2‐way ANOVA with Tukey post hoc test). The amount of free PAA was approximately 15‐fold higher in female than in male mice (Figure 1C through 1E). PAA levels in male mice were comparable with those observed in individuals with CVD,^21^ whereas levels in female mice more closely resembled those observed in individuals with end‐stage renal failure.^18^

Only male mice treated with PAA had slightly higher body weights by the end of the exposure (Figure 1F), without significantly affecting their body composition (percent fat or lean mass; Figure S1A and S1B). PAA‐treated male mice also had higher fasting glucose, homeostatic model assessment for insulin resistance, and plasma triglycerides, whereas no differences were observed in female mice (Figure S1C; Figure 1G and 1H). The only significant sex‐specific difference was that PAA‐treated male mice had higher homeostatic model assessment for insulin resistance than PAA‐treated female mice (*P*<0.0001, 2‐way ANOVA with Tukey post hoc test). Circulating levels of total, LDL/VLDL, and HDL cholesterol, LDL‐to‐HDL cholesterol ratio, and circulating cytokines were not affected by the PAA treatment in either sex (Figure 1I through 1K; Figure S1D through S1L). Although PAA did not alter overall cholesterol levels, total cholesterol was significantly higher in male than female mice in both control and PAA‐treated groups (both *P*<0.0001, 2‐way ANOVA with Tukey post hoc test). LDL/VLDL cholesterol levels were also higher in male than female mice but only in PAA‐treated animals (*P*=0.0002, 2‐way ANOVA with Tukey post hoc test). HDL cholesterol levels were higher in male versus female control mice (*P*<0.0001, 2‐way ANOVA with Tukey post hoc test) as well as PAA‐treated groups (*P*=0.003, 2‐way ANOVA with Tukey post hoc test).

**Figure 2:**
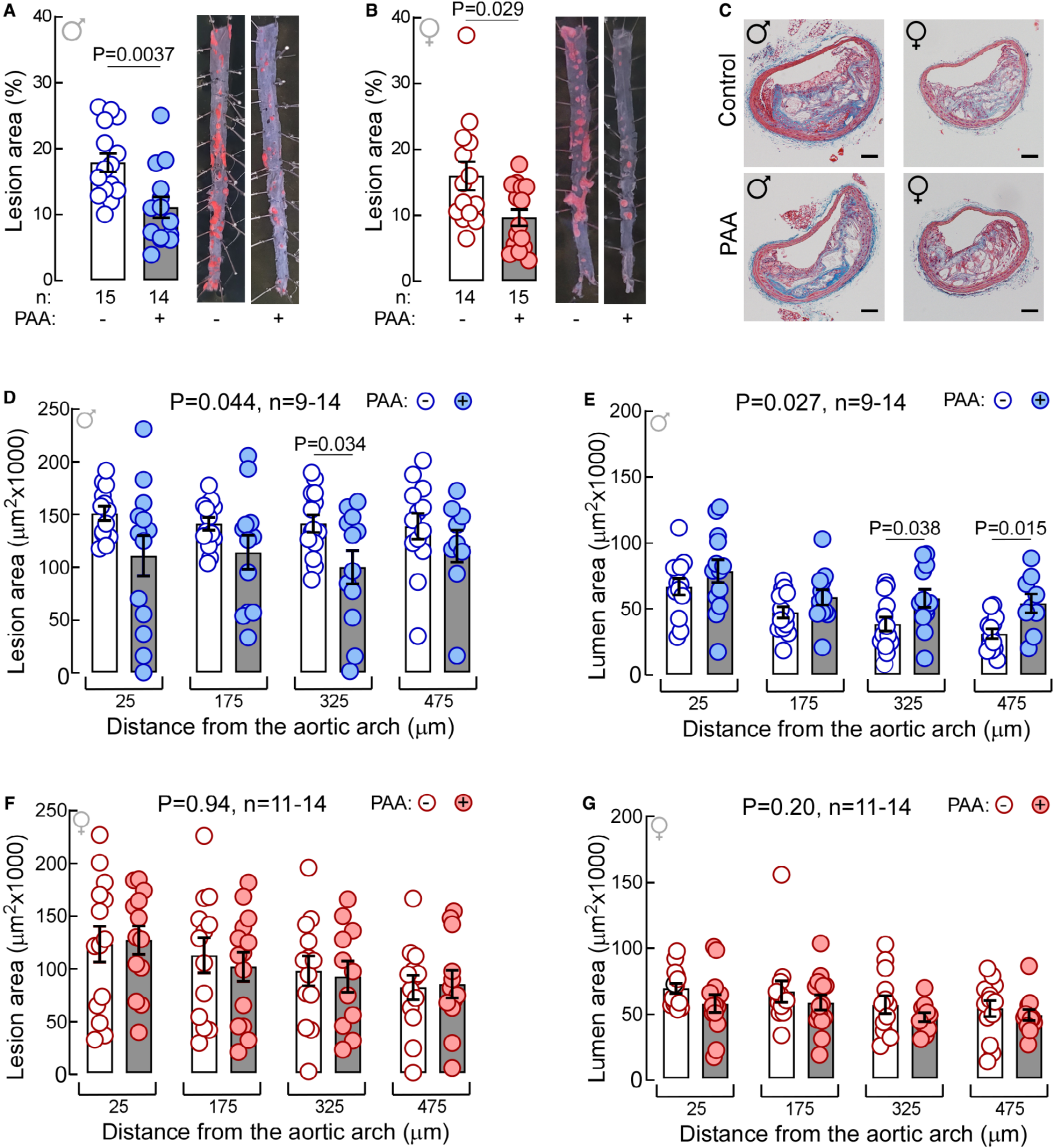
Dietary provision of PAA attenuates atherosclerosis burden in *Apoe* ^−/−^ mice fed a Western diet. **A** and **B**, Sudan IV *en face* staining and quantification of lesion area in aortas of male (blue) and female (red) *Apoe* ^−/−^ mice with (● symbols) and without (○ symbols) dietary provision of PAA. **C**, Masson Trichrome staining of the representative BCA cross‐sections. Scale bars=100 μm. **D** through **G**, Quantitative analysis of atherosclerotic lesion area and lumen area according to Masson Trichrome staining of atherosclerotic lesion cross‐sections within the control (○ symbols) and PAA‐treated (● symbols) BCAs of male (blue) and female (red) mice. Data were analyzed by Mann‐Whitney test (**A** and **B**) or mixed‐model ANOVA followed by Tukey post hoc test (**D** through **G**). Values are mean±SEM; n=9–14, as indicated. See also Figure S2. BCA indicates brachiocephalic artery; and PAA, phenylacetic acid.

Interestingly, *en face* Sudan IV staining showed that chronic PAA supplementation through drinking water reduced lipid burden by approximately 40% within thoracic and abdominal aortas of both male (Figure 2A) and female mice (Figure 2B). PAA‐treated male mice also had smaller lesions and larger lumen areas within defined regions of the brachiocephalic arteries compared with control mice (Figure 2C through 2E). In female mice, these differences were not statistically significant (Figure 2C, 2F, and 2G). Areas within the external elastic lamina, internal elastic lamina, or media were unchanged in both sexes (Figure S2A through S2F), indicating no evidence of vascular wall remodeling in response to PAA supplementation. Consistent with reduced plaque areas, PAA‐treated male mice had smaller necrotic core areas within the lesions (Figure S2G), whereas no differences were observed in female mice (Figure S2H).

**Figure 3:**
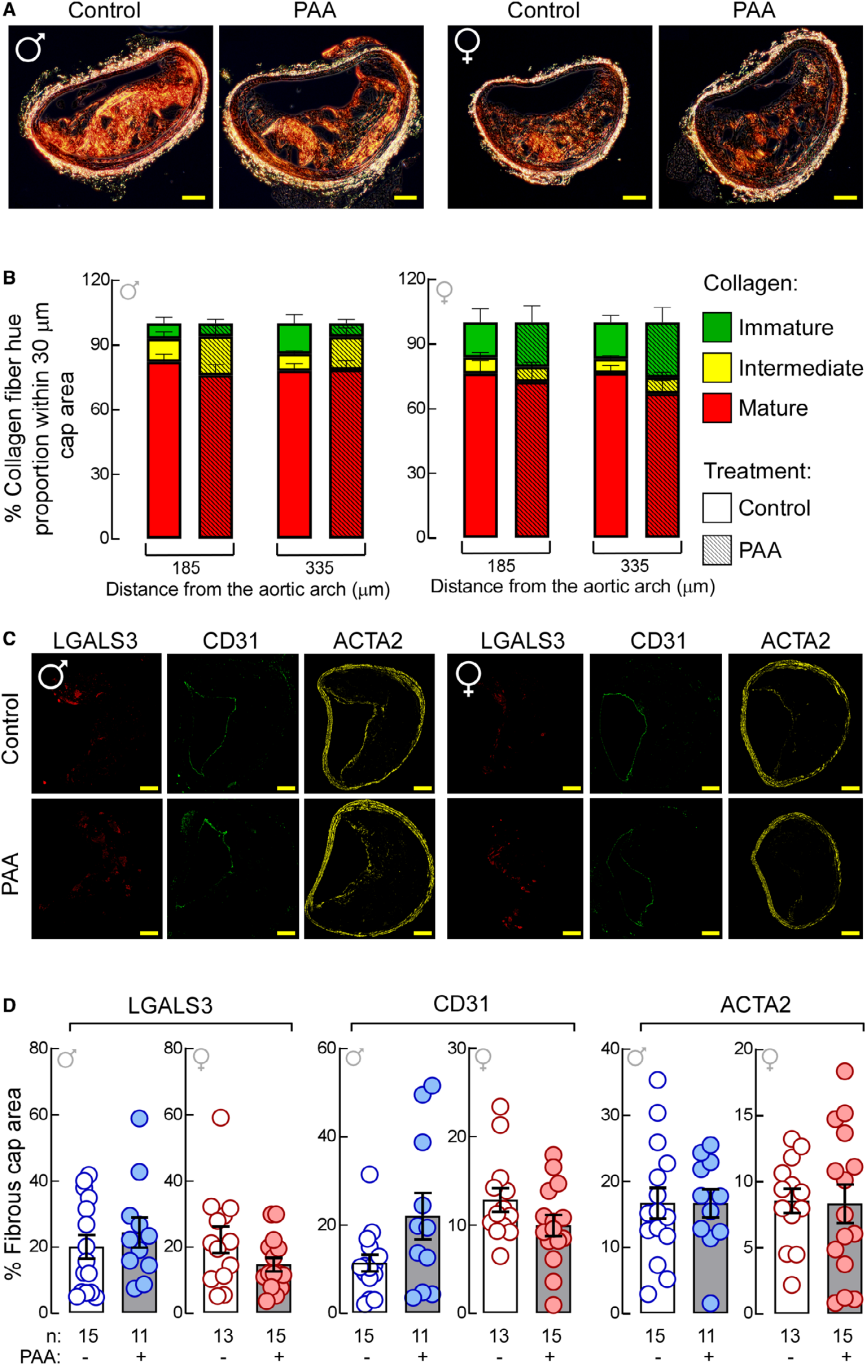
Dietary provision of PAA does not affect indices of plaque stability. **A**, PicroSirius Red staining of representative BCA cross‐sections of control and PAA‐treated male and female *Apoe* ^−/−^ mice. Scale bar=100 μm. **B**, Assessment of collagen fiber hue variations based on PicroSirius Red staining followed by polarized light microscopy. The ratio between immature (green), intermediate (yellow), and mature (red) collagen fibrils within the 30 μm fibrous cap area. **C**, Representative images of immunofluorescence staining for LGALS3 (red), CD31 (green), and ACTA2 (yellow); control and PAA‐treated male and female *Apoe* ^−/−^ mice. Scale bar=100 μm. **D**, LGALS3, CD31, and ACTA2 positive percent of fibrous cap area based on immunofluorescence staining within the control (○ symbols) and PAA‐treated (● symbols) BCAs of male (blue) and female (red) *Apoe* ^−/−^ mice. Data were analyzed by mixed‐model ANOVA, followed by Šídák post hoc test (**B**) or Mann‐Whitney test (**D**). Values are mean±SEM; n=11–14, as indicated. See also Figure S3. ACTA2 indicates smooth muscle α‐actin 2; BCA, brachiocephalic artery; CD31, platelet endothelial cell adhesion molecule‐1; LGALS3, galectin‐3; and PAA, phenylacetic acid.

We also examined indices of plaque stability, including collagen maturation and changes in the frequency of LGALS3, ACTA2, and CD31‐positive cells within the lesions and 30 μm fibrous cap area. No significant treatment‐related differences were observed among the experimental groups (Figure 3A through 3D; Figure S3A and S3B). However, the percentage of CD31‐stained fibrous cap and plaque area was higher in male versus female PAA‐treated mice (*P*=0.011 and *P*=0.025, respectively, 2‐way ANOVA with Tukey post hoc test). The ACTA2‐stained fibrous cap area was also higher in both control and PAA‐treated male versus female mice (*P*=0.012 and *P*=0.014, respectively, 2‐way ANOVA with post hoc Tukey test), but not in the whole plaque area.

Taken together, these data indicate that dietary PAA mediates cholesterol‐independent atheroprotective responses in *Apoe* ^−/−^ mice by decreasing plaque size without detectable changes in plaque stability or intraplaque cell composition.

### PAA Affects Gut Microbial Composition Without Significantly Changing the Output of Metabolites Associated With Atherosclerosis

**Figure 4:**
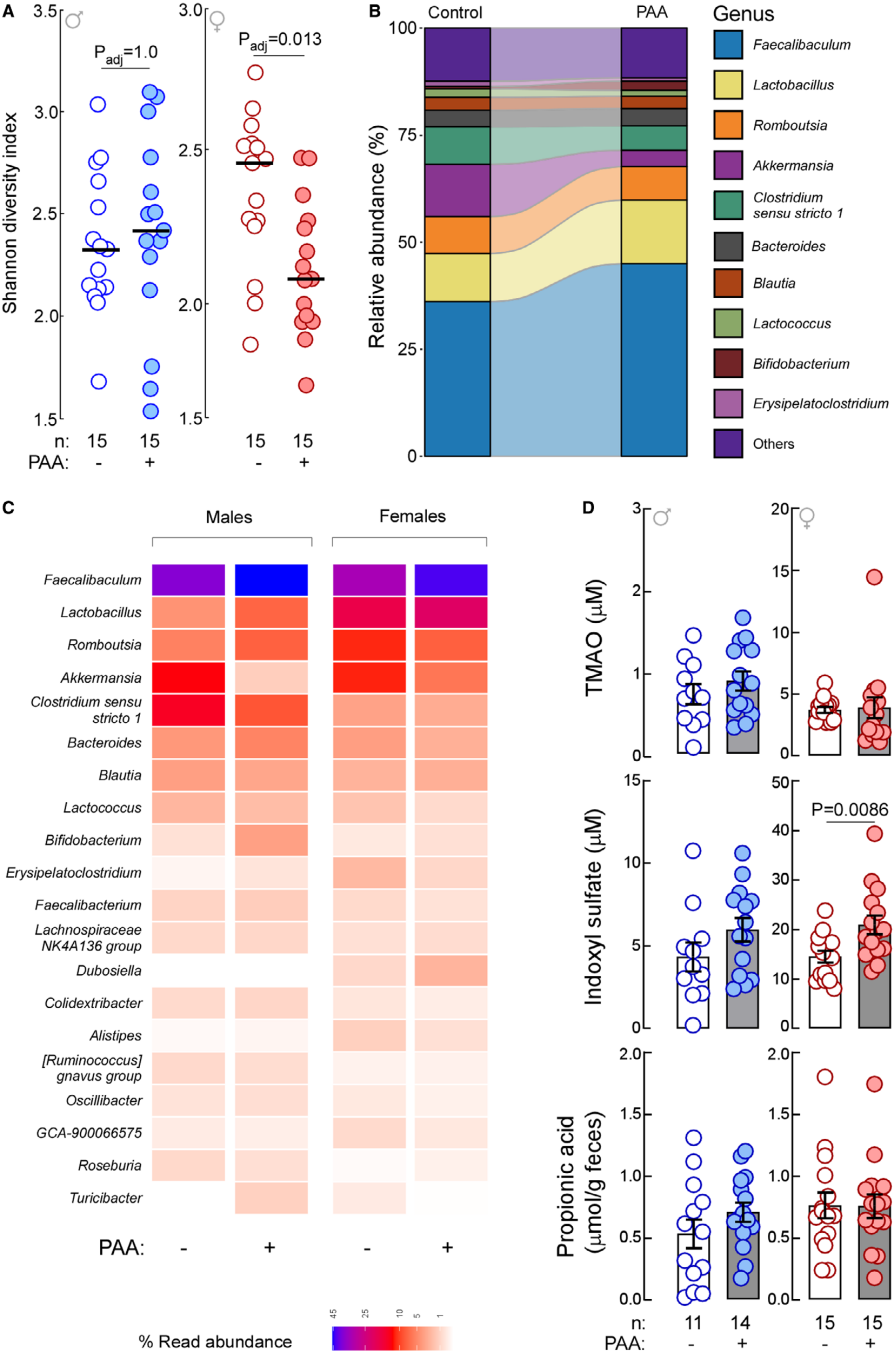
PAA affects gut microbial composition without changing the output of metabolites associated with atherosclerosis. **A**, Changes in the Shannon diversity index of male (blue) and female (red) *Apoe* ^−/−^ mice with dietary provision of PAA (● symbols) vs controls (○ symbols). **B**, Changes in the relative abundance of microbe genera in all control and PAA‐treated *Apoe* ^−/−^mice. **C**, Changes in the relative abundance of microbe genera among *Apoe* ^−/−^ male and female control and PAA‐treated mice. **D**, Circulating levels of TMAO (top), indoxyl sulfate (middle), and fecal levels of propionic acid (bottom) within the control (○ symbols) and PAA‐treated (● symbols) male (blue) and female (red) *Apoe* ^−/−^ mice. The Mann‐Whitney test (**D**) was used for statistical analysis; all other analyses are detailed in the Methods section. Values are mean±SEM; n=11–14 as indicated. See also Figure S4. GCA indicates GeneBank genome assembly accession number; PAA, phenylacetic acid; and TMAO, trimethylamine *N*‐oxide.

Because changes in gut microbial composition are associated with atherosclerosis progression, we next evaluated the effects of PAA treatment on gut microbial composition by 16S rDNA sequencing of fecal samples. Dietary PAA supplementation resulted in a clear separation of gut microbiota clusters (Figure S4A). Overall microbial diversity was unchanged in male mice but decreased in female mice (Figure 4A). At the genus level, PAA treatment increased the relative abundance of *Faecalibaculum*, *Lactobacillus*, and *Bifidobacterium*, while decreasing *Romboutsia*, *Akkermansia*, *Clostridium sensu stricto 1*, *Bacteroides*, *Lactococcus*, and *Erysipelatoclostridium* (Figure 4B). When stratified by sex, SCFA‐producing *Faecalibaculum* was increased in both sexes, along with *Lactobacillus* and *Bifidobacterium. Akkermansia*, a genus of bacteria generally associated with positively impacting metabolic health, decreased to a greater extent in male mice, whereas *Bifidobacterium*, which has beneficial anti‐inflammatory properties, increased more in male than female mice following PAA treatment (Figure 4C). Despite the differences in gut microbial composition between control versus PAA‐treated groups, we did not observe greater differences in levels of microbially produced metabolites clinically and mechanistically linked to CVD in humans or animal models (microbial community output). However, several CVD‐associated gut microbial metabolites were overall higher in female than male mice. Specifically, TMAO, an atherosclerosis contributing gut microbial metabolite, was not affected by PAA treatment in either sex (Figure 4D, top panel), but its levels were higher in female than male mice both in control and PAA‐treated groups (both *P*<0.001, 2‐way ANOVA with Tukey test post hoc test). Female mice also had overall higher levels of indoxyl sulfate (both *P*<0.0001, 2‐way ANOVA with Tukey post hoc test), a gut microbially derived uremic toxin involved in atherosclerosis‐related pathological processes,^37^ than male mice, and PAA treatment slightly worsened its levels in female mice (Figure 4D, mid panel). Other uremic toxins, such as *p*‐cresol sulfate and indoxyl glucuronide, were unchanged in both sex (Figure S4B and S4C), and we did not observe any worsening of kidney function in either sex (Figure S4D). PAA treatment also increased circulating levels of 4HPA in both sexes (Figure S4E), with overall higher levels in PAA‐treated female than male mice (*P*<0.0001, 2‐way ANOVA with Tukey post hoc test). 4HPA levels strongly correlated with PAA concentrations (Figure S4F). Propionic acid, a gut microbially derived SCFA with cholesterol‐lowering properties,^4^ was unchanged across experimental groups and sexes (Figure 4D, bottom panel). Similarly, other measured SCFAs were also unaffected by the PAA treatment; however, lactic acid levels were higher in female than male mice, in both control (*P*=0.021, 2‐way ANOVA with Tukey post hoc test) and PAA‐treated (*P*=0.001, 2‐way ANOVA with Tukey post hoc test) groups (Figure S4G through S4I).

Collectively, dietary provision of PAA altered gut microbial composition and some features of microbial community metabolic outputs. Although these changes may contribute to sex‐specific differences in atherosclerotic burden observed with PAA supplementation, they do not fully explain the overall reduction in the atherosclerotic lesion size.

### PAA Impacts Carnitine Homeostasis

An increase in fasting glucose, insulin, and circulating triglycerides in male mice, accompanied by the concomitant reduction in atherosclerotic burden across multiple vascular beds, suggests a metabolic imbalance in fat metabolism and distribution.

**Figure 5:**
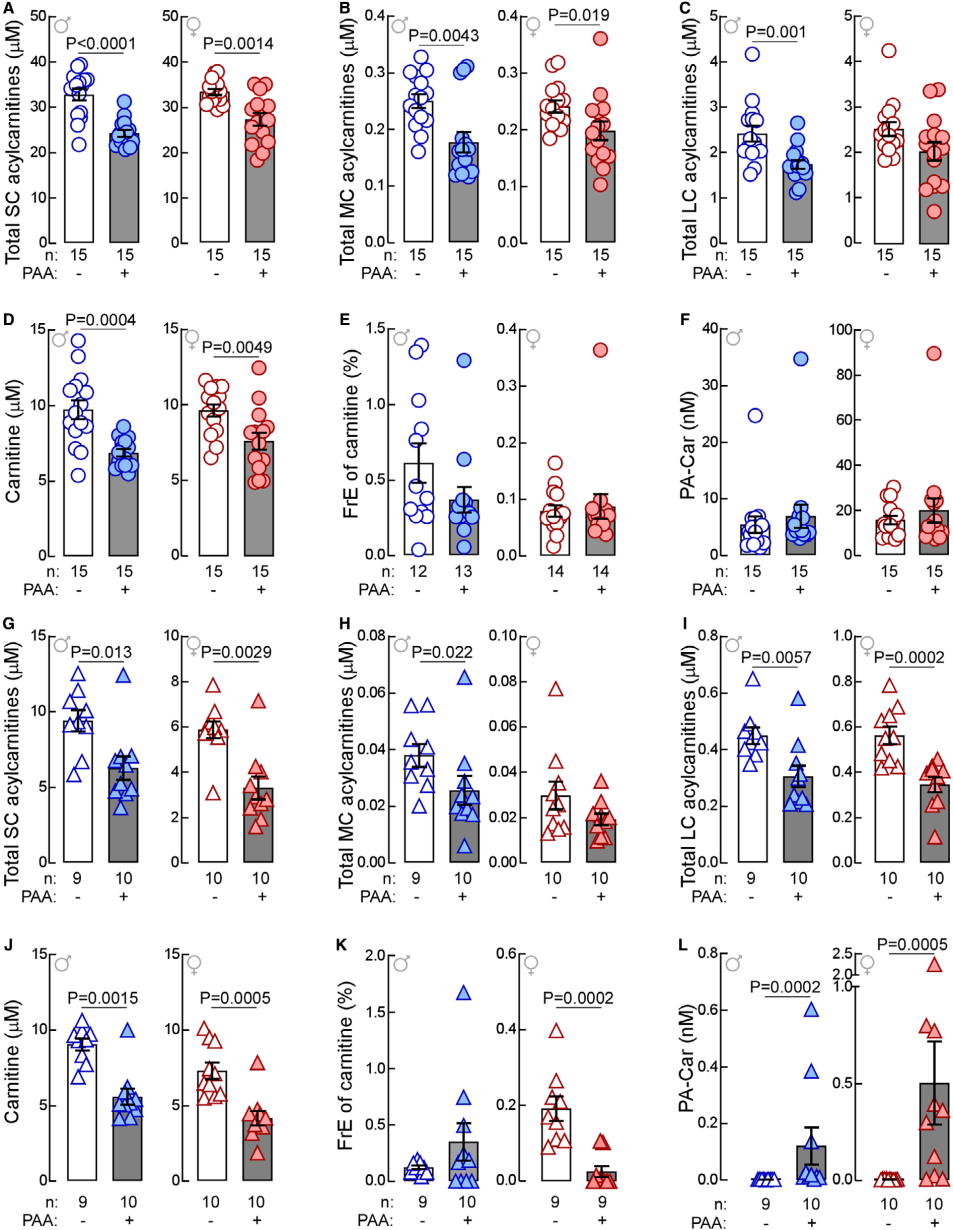
PAA decreases circulating levels of the total carnitine pool in *Apoe* ^−/−^ and WT mice. Circulating levels of total SC acylcarnitines (**A** and **G**), MC acylcarnitines (**B** and **H**), LC acylcarnitines (**C** and **I**), and carnitine (**D** and **J**). FrE of carnitine (**E** and **K**), and circulating levels of PA‐Car (**F** and **L**) in control (○ symbols) and PAA‐treated (● symbols) of male (blue) and female (red) *Apoe* ^−/−^ (circles) and WT (triangles) mice. The Mann‐Whitney test was used for statistical analysis. Values are mean±SEM; n=9–15, as indicated. See also Figure S5. FrE indicates fractional renal excretion; LC, long‐chain; MC, medium‐chain; PAA, phenylacetic acid; PA‐Car, phenylacetyl‐carnitine; SC, short‐chain; and WT, wild‐type.

Profiling circulating acylcarnitines is commonly used to diagnose inborn errors of fatty acid oxidation. Recently, acylcarnitine profiles have been recognized as markers of metabolic disorders associated with insulin resistance and obesity, as well as those associated with heart failure and CAD.^38^, ^39^ Therefore, we measured their levels in fasting plasma from the experimental groups using a quantitative stable isotope dilution LC–MS/MS method. As depicted in Figure 5A through 5C, PAA treatment reduced levels of total short‐chain and medium‐chain acylcarnitines in both sexes, as well as long‐chain acylcarnitines significantly in male mice. No sex‐specific differences were observed in the magnitude of reduction. A decrease in acylcarnitine levels due to improved fatty acid β‐oxidation is usually accompanied by an increase in free carnitine. However, dietary PAA caused an overall reduction in free carnitine (Figure 5D). Lower circulating carnitine levels were not due to its increased kidney excretion, because the fractional renal excretion of carnitine was not elevated in the PAA experimental groups compared with the controls (Figure 5E). Because carnitine scavenges acyl residues derived from intermediary coenzyme A metabolism beyond those arising from fatty acids,^40^ we hypothesized that the observed secondary carnitine deficiency resulted from increased production of PA‐Car from PAA (Figure S5A), as previously suggested.^41^ Although PA‐Car increased with PAA supplementation in plasma from fed animals (Figure S5B), the increase could not account for the overall loss of the carnitine pool. Moreover, levels of PA‐Car in fasted animals did not differ significantly between the treatment groups (Figure 5F), although we observed higher PA‐Car levels in female than male mice, but only after the PAA treatment (*P*=0.020, 2‐way ANOVA with Tukey post hoc test).

**Figure 6:**
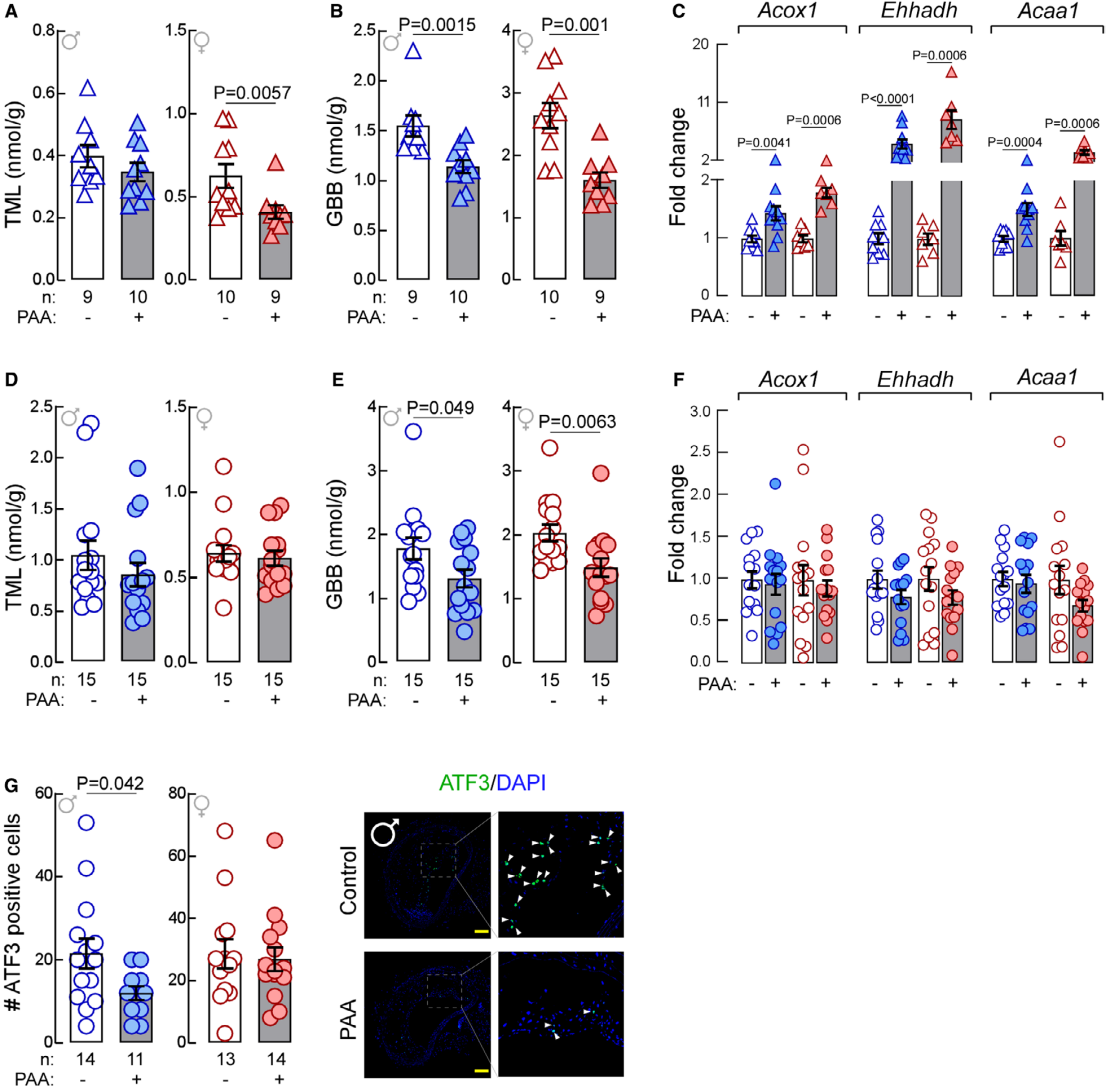
PAA increases the expression of genes associated with peroxisomal lipid metabolism. **A**, **B**, **D**, and **E**, Hepatic levels of TML and GBB in control (○ symbols) and PAA‐treated (● symbols) male (blue) and female (red) WT (triangle) and *Apoe* ^−/−^ (circle) mice. **C** and **F**, Hepatic expression of indicated genes in control (○ symbols) and PAA‐treated (● symbols) male (blue) and female (red) WT (triangles) and *Apoe* ^−/−^ (circles) mice. **G**, Number of ATF3‐positive cells within atherosclerotic lesions of BCAs in control (○ symbols) and PAA‐treated (● symbols) male (blue) and female (red) *Apoe* ^−/−^ mice (circle) and representative images of immunofluorescence staining for ATF3 (green, arrowhead symbols) and DAPI (blue) in BCAs isolated from male mice. Scale bar=100 μm. The Mann‐Whitney test was used for statistical analysis. Values are mean±SEM; n=9–15, as indicated. See also Figure S6. *Acaa1* indicates gene encoding for acetyl‐coenzyme A acyltransferase 1; *Acox1*, gene encoding for acyl‐coenzyme A oxidase 1; ATF3, activating transcription factor 3; BCA, brachiocephalic artery; *Ehhadh*, gene encoding for enoyl‐coenzyme A hydratase/3‐hydroxyacyl coenzyme A dehydrogenase; GBB, γ‐butyrobetaine; PAA, phenylacetic acid; TML, trimethyllysine; and WT, wild‐type.

To validate these findings (ie, to test if PAA also reduces the carnitine pool in a nonatherosclerotic mouse model) and to further investigate the role of PAA's effect on carnitine homeostasis, PAA was also provided in drinking water to male and female WT mice. Similar to findings in *Apoe* ^−/−^ mice, dietary provision of PAA raised circulating levels of phenylacetylglycine, phenylacetylglutamine, and free PAA (Figure S5C through S5E). In WT mice, PAA did not alter body weight, food consumption, body composition, or fasting glucose in either sex, although liver weight increased in PAA‐treated female mice (Figure S5F through S5K). Matching the atherosclerotic mice, the dietary provision of PAA reduced the total circulating carnitine pool in WT mice without increasing urinary excretion or inducing PA‐Car production to an extent that would explain the overall carnitine loss (Figure 5G through 5L). Notably, short‐chain acylcarnitines were significantly higher in male than female mice (*P*=0.001 for control and *P*=0.007 for PAA‐treated mice; 2‐way ANOVA with Tukey post hoc test). Next, we checked if the reduced carnitine pool is a result of a decrease in its endogenous synthesis. Endogenous carnitine is synthesized in the liver from trimethyllysine via GBB (Figure S6A). PAA did not affect the expression of genes involved in carnitine biosynthesis in WT mice (Figure S6B), but it reduced hepatic trimethyllysine levels in female mice and GBB levels in both sexes (Figure 6A and 6B). We also note that GBB levels were overall higher in female than male WT control mice (*P*<0.0001, 2‐way ANOVA with Tukey post hoc test).

Because carnitine is essential for mitochondrial fatty acid transport, we evaluated whether the reduced carnitine pool altered expression of genes in the carnitine shuttle. PAA treatment had mixed effects on carnitine‐shuttle gene expression in WT animals, suggesting that PAA did not lead to general improvement in β‐oxidation (Figure S6C and S6D). However, PAA markedly increased expression of genes encoding acyl‐coenzyme A oxidase 1 (*Acox1*), enoyl‐coenzyme A hydratase/3‐hydroxyacyl coenzyme A dehydrogenase (*Ehhadh*), and acetyl‐coenzyme A acyltransferase 1 (*Acaa1*) in both sexes, indicating activation of peroxisomal lipid oxidations (Figure 6C; Figure S6C).

In *Apoe* ^−/−^ mice, hepatic trimethyllysine levels were unchanged by PAA (Figure 6D), whereas GBB was reduced in both sexes, consistent with findings in WT animals (Figure 6E). Expression of fatty acid metabolism genes in *Apoe* ^−/−^ livers did not differ between treatment groups (Figure 6F; Figure S6E). Notably, livers from *Apoe* ^−/−^ mice were collected after overnight fasting, which both enhances expression of genes involved in lipid metabolism and reduces circulating PAA and its downstream metabolites.

Peroxisomes, beyond breaking down fatty acids, also play an important role in reducing oxidative and endoplasmic reticulum stress. To test this, we examined the expression of ATF3, a stress‐response protein upregulated during endoplasmic reticulum stress. We observed a lower number of ATF3‐positive cells within the lesions of PAA‐treated *Apoe* ^−/−^ male mice compared with the control group (Figure 6G). In female mice, ATF3 levels did not differ significantly between treatment and control groups (Figure 6G).

Taken together, dietary provision of PAA reduced the total (free + ester‐bound) carnitine pool in both *Apoe* ^−/−^ and WT mice, increased the expression of genes encoding enzymes involved in peroxisomal fatty acid oxidation in WT mice, and lowered ATF3 within the atherosclerotic lesions of PAA‐treated *Apoe* ^−/−^ male mice.

## DISCUSSION

Here we show that dietary provision of PAA, despite increasing circulating levels of phenylacetylglycine to the amounts equivalent to the human PAA‐analogue phenylacetylglutamine associated with CVD,^21^, ^22^, ^23^ reduced atherosclerotic burden within descending and abdominal aortas in both sexes and within brachiocephalic arteries in male mice, without affecting indices of plaque stability. Differences in atherosclerotic burden within brachiocephalic arteries in male versus female mice could be in part due to the higher residual PAA levels in female than in male mice.

It has been shown that PAA can induce endothelial cell senescence *in vitro*.^16^ Additionally, spatially resolved metabolic analysis of human atheroma observed enrichment of PAA within unstable plaques.^42^ It is unclear whether this enrichment reflects elevated circulating PAA in those individuals or whether PAA was locally produced by the microbes residing within plaques. Namely, microbes of the oral and gut origin have been identified within human atherosclerotic lesions,^43^ with some of these are known PAA producers.^44^

In our study, we also observed reduced gut microbial diversity in female mice following PAA ingestion. Levels of several atherosclerosis‐associated metabolites, such as TMAO and indoxyl sulfate, were significantly higher in female than male mice, offering additional explanation for sex‐specific differences. Dietary PAA also elevated circulating 4HPA. 4HPA is a microbial flavonoid‐ and tyrosine‐derived catabolite known to reduce diet‐induced cardiometabolic disease burden in mice.^45^ Because the 4HPA levels in our study were substantially higher than those regularly observed in mice, even in mice fed diets enriched in flavonoids, we hypothesize that 4HPA is produced directly from PAA by the host enzymes. This is supported by the strong correlation between 4HPA and PAA levels and is consistent with enhanced peroxisomal activation following PAA treatment.

PAA treatment further reduced circulating free carnitine and its acyl‐adducts in both sexes. The role of carnitine in atherosclerosis progression remains unclear, because several published studies have yielded conflicting findings. The main function of carnitine is the transport of log‐chain fatty acids into the mitochondrial matrix for β‐oxidation and maintaining the acetyl‐coenzyme A/coenzyme A ratio. Furthermore, carnitine has antioxidant and anti‐inflammatory properties,^46^ making it a promising supplement in the treatment of CAD. A small randomized, placebo‐controlled trial showed that 12‐week carnitine supplementation in patients with CAD increased HDL cholesterol and apolipoprotein A1, and caused a slight, but not significant, decrease in triglycerides.^47^ Conversely, 6‐month carnitine supplementation to individuals with metabolic syndrome in a randomized controlled trial did not impact total carotid plaque size; however, there was a greater progression of carotid stenosis with carnitine supplementation.^48^ Moreover, suppression of carnitine biosynthesis via a GBB dioxygenase inhibitor decreased the carnitine pool in the aortic tissues and overall atherosclerotic burden in *Apoe* ^−/−^ mice.^49^, ^50^ Furthermore, macrophage‐specific deletion of a gene encoding for carnitine palmitoyltransferase 2 elevated long‐chain acylcarnitine, reduced short‐chain and medium‐chain acylcarnitines, and exacerbated atherosclerosis in the *Apoe* ^−/−^ mice.^51^ Additional studies suggest that dietary carnitine might accelerate atherosclerosis due to its conversion to the proatherogenic gut microbially metabolite TMAO,^52^ which may explain different outcomes across carnitine supplementation studies. In our study, PAA did not affect circulating TMAO, suggesting that the observed reduction in atherosclerosis burden was not mediated through TMAO. We also showed that dietary provision of PAA reduced the carnitine pool in nonatherosclerotic WT mice without altering expression of genes involved in carnitine biosynthesis; instead, PAA lowered levels of the biosynthetic precursors trimethyllysine and GBB, known to limit the rate of carnitine biosynthesis.^53^, ^54^

PAA is approved for treating urea cycle disorders, rare genetic disorders characterized by hyperammonemia. Although the impact of PAA therapy on carnitine levels in those individuals is largely unknown, elevated PAA and carnitine deficiency frequently co‐occur in phenylketonuria.^55^, ^56^, ^57^ Although this has been attributed to reduced carnitine dietary intake, our study suggests that PAA directly impacts host carnitine homeostasis.

We also observed that PAA upregulated genes involved in peroxisomal lipid metabolism in WT mice. Prior studies have shown that administration of 4‐phenylbutyric acid, a pharmacological inducer of peroxisome biogenesis,^58^ alleviated atherosclerosis in several animal models.^59^, ^60^ Similarly to PAA, 4‐phenylbutyric acid is used to treat urea cycle disorders and is rapidly converted to phenylbutyryl‐coenzyme A and then to PAA,^61^ suggesting that the benefits attributed to 4‐phenylbutyric acid may arise from its downstream metabolite. The beneficial effects of 4‐phenylbutyric acid were explained by its reduction of endoplasmic reticulum stress within atherosclerotic lesions, consistent with the lower ATF3 expression we observe in PAA‐treated male mice. ATF3 induction was also associated with atherosclerotic progression in humans.^62^ Because ATF3 is induced by various stress stimuli beyond the endoplasmic reticulum stress, reduced ATF may reflect additional mechanisms. A recent study showed that hepatic overexpression of ATF3 reduced atherosclerosis in different mouse models.^63^ In our study, we did not observe differences in hepatic *Atf3* gene expression among the treatment groups, and we did not observe major changes in liver cholesterol or bile acid metabolism (data not shown). We do note that lower levels of carnitine were also accompanied by lower levels of its synthetic precursors trimethyllysine and GBB. Because trimethyllysine is produced endogenously through the breakdown of proteins, lower hepatic levels of trimethyllysine could be a result of lower protein turnover.

Several clinical studies have shown strong associations between phenylacetylglutamine and CVD. Here we show that those associations are not primarily driven by an increase in atherosclerotic burden. Instead, one of the major drivers is phenylacetylglutamine/phenylacetylglycine‐mediated platelet hyperresponsiveness appears to be a key mechanism.^6^ Observed reduction in acylcarnitines can also be an additional contributor to the association of phenylacetylglutamine with thrombotic events, besides increased platelet hyperresponsiveness in the presence of known platelet agonists. Many acylcarnitines, particularly long‐chain acylcarnitines, are reduced in venous thrombosis, and they exhibit anticoagulant properties in clotting assays.^64^ A separate study associated increased levels of medium‐chain acylcarnitines with cardioembolic stroke and stroke recurrence, but without showing any direct effects on platelet function.^65^ In clinical studies, phenylacetylglutamine also showed strong associations with heart failure.^7^, ^8^ The adult heart relies heavily on fatty acid β‐oxidation, and alteration in carnitine metabolism could contribute to myocardial dysfunction. A meta‐analysis of randomized controlled trials involving patients with chronic heart failure showed that carnitine supplementation, although it did not significantly reduce all‐cause mortality between groups, improved left ventricular ejection fraction, stroke volume, and cardiac output compared with controls, as well as reduced serum basal natriuretic peptide and N‐terminal prohormone of basal natriuretic peptide levels.^66^

It is also important to note the limitations of our model. Although the *Apoe* ^−/−^ mouse model has well‐recognized limitations, including a distinct lipid profile, lack of plaque rupture and thrombosis, and differences in metabolite handling compared with humans, it remains a standard model for mechanistic studies of atherosclerosis and microbe–host interactions. Importantly, our observation that PAA treatment reduced the total carnitine pool in both *Apoe* ^−/−^ and nonatherosclerotic WT mice suggests that these metabolic effects are not artifacts of the *Apoe* knockout model, but rather reflect broader host responses, underscoring the need for future validation in complementary models. Although dietary PAA increased phenylacetylglutamine, mice predominantly formed phenylacetylglycine from PAA, and differences between human and murine metabolism may explain discrepancies with clinical data. The only potential target for phenylacetylglutamine described to date is the β2‐adrenergic receptor, in which phenylacetylglutamine acts as a negative allosteric modulator and induces a negative inotropic effect on isolated mouse cardiomyocytes, but only in the presence of β‐adrenergic receptor agonist isoproterenol.^67^ In the same study, phenylacetylglycine, although not tested thoroughly in all *in vitro* screens as with phenylacetylglutamine, showed a negative inotropic effect to the same extent on mouse cardiomyocytes as phenylacetylglutamine, suggesting that they both act through a similar mechanism.^67^ Additionally, phenylacetylglycine and phenylacetylglutamine had the same effect on thrombus formation in mice,^6^ further suggesting similar biological effects and mechanisms of action. However, a recent study showed that only phenylacetylglutamine, but not phenylacetylglycine, caused heart failure phenotypes in mice,^20^ suggesting that these 2 metabolites have the same but also differential effects on the host. Because our model exposes animals to a mixture of PAA‐derived metabolites, direct intraperitoneal delivery of individual compounds is limited by rapid mouse kidney clearance (intraperitoneally injected phenylacetylglutamine and phenylacetylglycine clear within 30 minutes^6^, ^7^), seems an unsuitable model for studying long‐term subjection to those metabolites. However, a recent study with repeated phenylacetylglutamine intraperitoneal injections twice daily for 20 days induced elevated left ventricular end‐diastolic filling pressure and reduced plasma and tissue nitrite levels,^20^ suggesting that this could be a potential model for testing the effects of chronic phenylacetylglutamine exposure.

In conclusion, these results provide new insights into how gut‐microbial metabolism of phenylalanine influences host lipid and carnitine metabolism and atherosclerosis progression. Our findings suggest that clinical associations between phenylacetylglutamine and CVD risk are not primarily driven by increased atherosclerotic burden. Future studies should assess effects of individual metabolites, test the impact of PAA in the absence of glycine, and incorporate additional models such as LDL receptor knockout mice or humanized systems.

## Sources of Funding

This work is supported by grants from the National Institutes of Health (R01HL160747 to I.N. and R01HL150193 to O.A.C.) as well as Cleveland Clinic internal funding to I.N.

## Disclosures

None.

## Supporting information

Data S1 Supplemental Methods
Table S1
Figures S1–S6

ARRIVE Checklist
