# Supplementary material for: Phenylacetic Acid, a Gut Microbially Produced Metabolite, Reduces Atherosclerosis Burden and Impacts Host Lipid Homeostasis
Source: J Am Heart Assoc. 2026 Jul 10;15(14):e047044. doi: 10.1161/JAHA.125.047044 (PMC13477316; doi:10.1161/JAHA.125.047044)
Supplement: Supplementary file 2 [file JAH3-15-e047044-s002.pdf]

SUPPLEMENTAL MATERIAL

Data S1. Supplemental Methods

Sample size, inclusion and exclusion criteria

Both male and female mice were included in the study and analyzed separately. A sample size of 13 *Apoe*$^{-/-}$ mice per treatment group for each sex was selected to provide 95% power to detect a 40% difference in lipid deposition, as measured by Sudan IV en face staining (primary outcome) of the whole aorta after 18 weeks of Western diet feeding. Two additional mice were added to each group to account for potential mortality. A sample size of 10 WT mice per treatment group for each sex was selected to provide 95% power to detect a 30% difference in carnitine pools (primary outcome).

All animals and all collected samples were included in the analyses except in the following situations: Sudan IV staining: One aorta from a female control animal was identified as a statistical outlier (77.2% Sudan IV–positive lesion area), and another aorta was excluded because it was damaged during isolation. Morphometric and immunohistochemical analysis of brachiocephalic arteries: Samples were excluded if the lumen boundary was not clearly visible or if the lesion shifted or became dislocated during tissue processing. Blood and urine collection: In some animals, insufficient blood or urine volume was obtained via saphenous vein sampling or urine collection, and these samples could not be used in the analyses.

Randomization and strategy used to minimize potential confounders

Randomization was not used to assign experimental units. Instead, mice were randomly allocated to cages by personnel who were blinded to the study design. After allocation, mice received treatment through drinking water; at this stage, personnel were no longer blinded to the treatment groups. All mouse cages were changed, and fresh water and food were provided on the same day for all animals.

Tissue collections occurred over multiple days. Each day included animals from all treatment groups, and mice were evenly distributed across collection days. During sample processing and data analysis, personnel were not blinded to the treatment groups.

Table S1. **Primer sequences used for real-time qPCR analysis.**

Gene name	Forward (5'–3')	Reverse (5'–3')
Tmlhe	CTGTGCCTTATGATGTTGTCCA	TGCCATGAAGTACTCGCCAG
Aldh9a1	CGTTGAGAATGCAAAGGCTG	ACTTCCCGTTGTTGATGGTC
Bbox1	TTGGCATCAATCTTGTCCTG	AGCTGACAAACGTGGTGAGA
Cpt1a	TTGGAAGTCTCCCTCCTTCA	GCCCATGTTGTACAGCTTCC
Cpt1b	GCTGCTTGCACATTTGTGTT	TGAGTGACTGGTGGGAAGAA
Cact	GGTGGCTGTCCAGACAAACT	TCCGTTTAAGAACCTCCTGG
Cpt2	TCTTCCTGAACTGGCTGTCA	GTACCCACCATGCACTACCA
CrAT	CTCCTGGGCTGGAGTAGATG	TTACAGAAGGGACTGGAGCG
Slc22a5	AAGACCTGCAGGAAGCTGAA	TCCTTGTTTTTCGTGGGTGT
Pparα	ACGATGCTGTCCTCCTTGATG	GCGTCTGACTCGGTCTTCTTG
Acox1	CCGCCTATGCCTTCCACT	ACCGCAAGCCATCCGACA
EHHADH	GTGGCTCTTGGAGGAGGACTA	TTCAACTGGGTCTGACTTTACAAC
Acaa1	GATGACCTCGGAGAATGTGG	CCTGAGACACGGTGATGGT
18S	CGGCTACCACATCCAAGGAA	AGCTGGAATTACCGCGGC

Figure S1. Dietary provision of PAA worsens fasting blood glucose levels in male *Apoe*$^{-/-}$ mice on a Western diet but it does not change levels of circulating inflammatory cytokines.

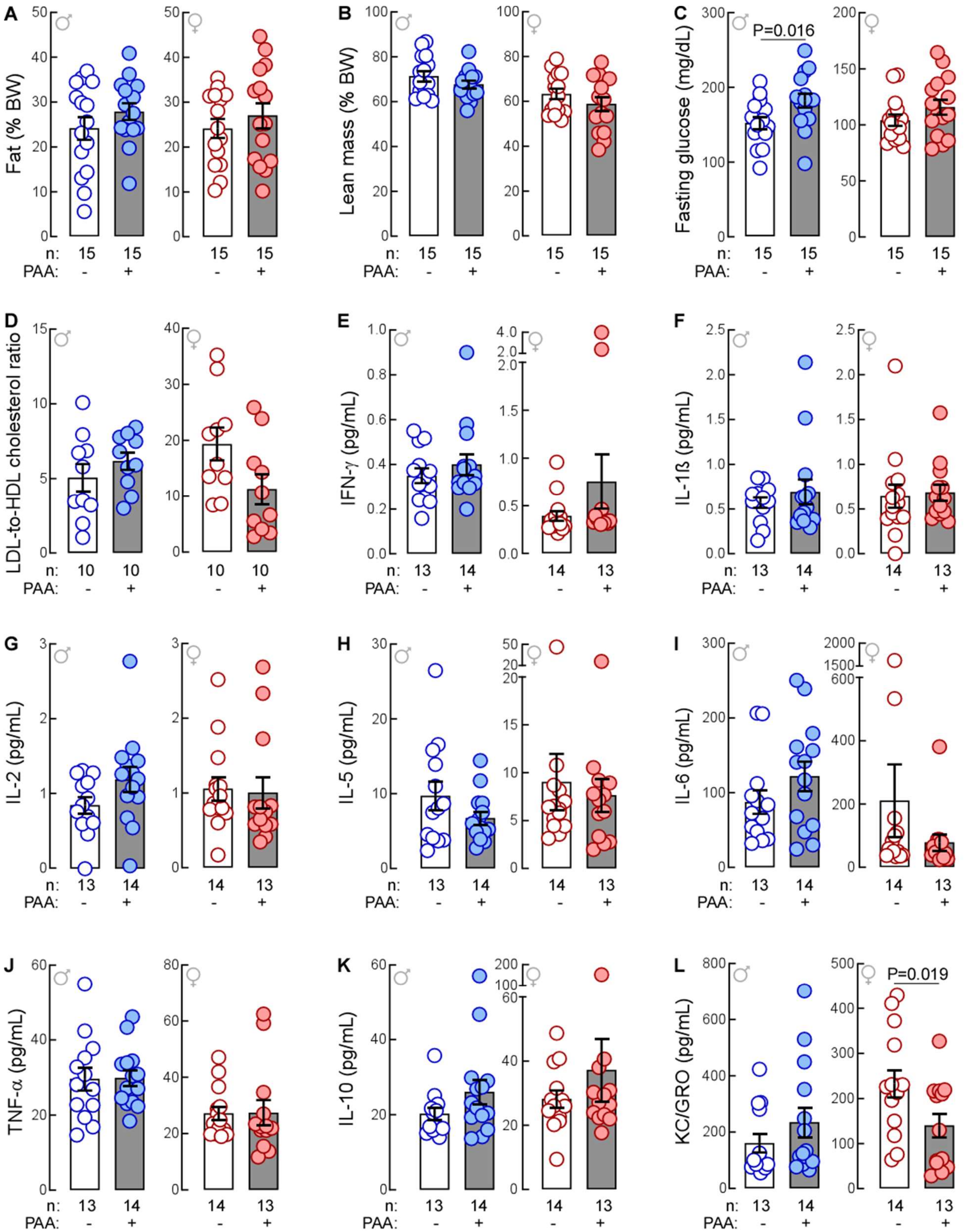

(**A,B**) Body composition established by EchoMRI in male (blue) and female (red) *Apoe*$^{-/-}$ mice on a Western diet with (full symbols) and without (open symbols) dietary provision of PAA. (**C**) Fasting blood glucose levels, circulating (**D**) LDL-to-HDL cholesterol ratio and (**E**-**L**) plasma cytokines. Data were analyzed by Mann-Whitney test. Values = mean ± SEM; n = 10-15 as indicated. BW indicates body weight; HDL, high-density lipoprotein; IFN-γ, interferon-γ; IL-1β, interleukin-1β; IL-2, interleukin-2; IL-5, interleukin-5; IL-6, interleukin-6; IL-10, interleukin-10; KC/GRO, keratinocyte-derived chemokine/growth-regulated oncogene; LDL, low-density lipoprotein; PAA, phenylacetic acid; and TNF-α, tumor necrosis factor-α.

Figure S1 is related to **Figure 1**.

Figure S2. Dietary provision of PAA reduces the severity of necrotic lesions in male *Apoe*$^{-/-}$ mice fed a Western diet.

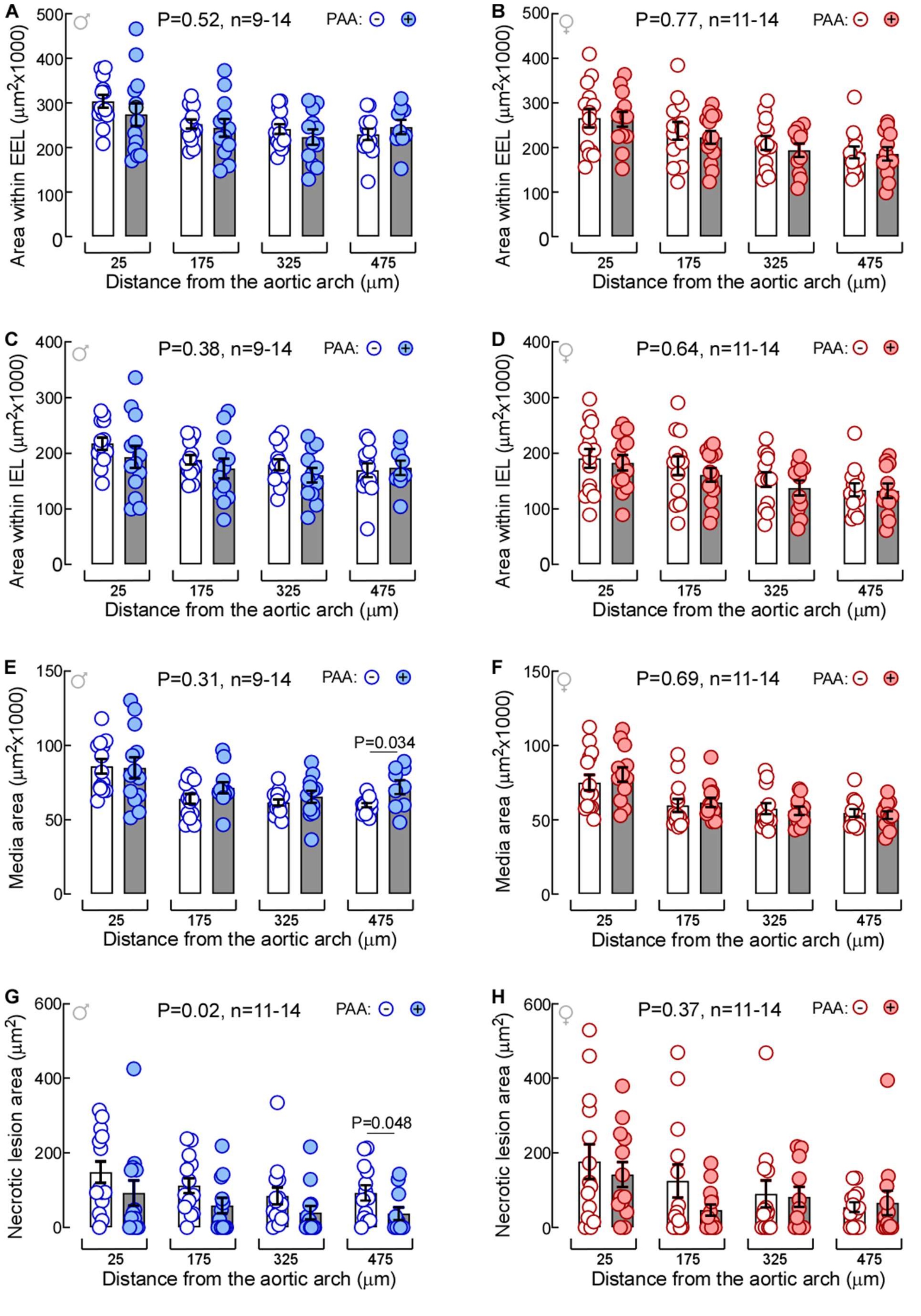

Quantitative analysis of areas within (**A, B**) EEL, (**C, D**) IEL, (**E, F**) media (EEL-IEL), and (**G, H**) necrotic lesion areas according to Masson's Trichrome staining of atherosclerotic lesion cross-sections within the control (open symbols) and PAA-treated (closed symbols) BCAs of male (blue) and female (red) *Apoe*$^{-/-}$ mice. Data were analyzed by mixed-model ANOVA, followed by Tukey's *post hoc* test. Values = mean ± SEM; n = 9-14. BCA indicates brachiocephalic artery; EEL, external elastic lamina; IEL, internal elastic lamina; and PAA, phenylacetic acid.

Figure S2 is related to **Figure 2**.

Figure S3. Dietary provision of PAA does not affect indices of plaque stability.

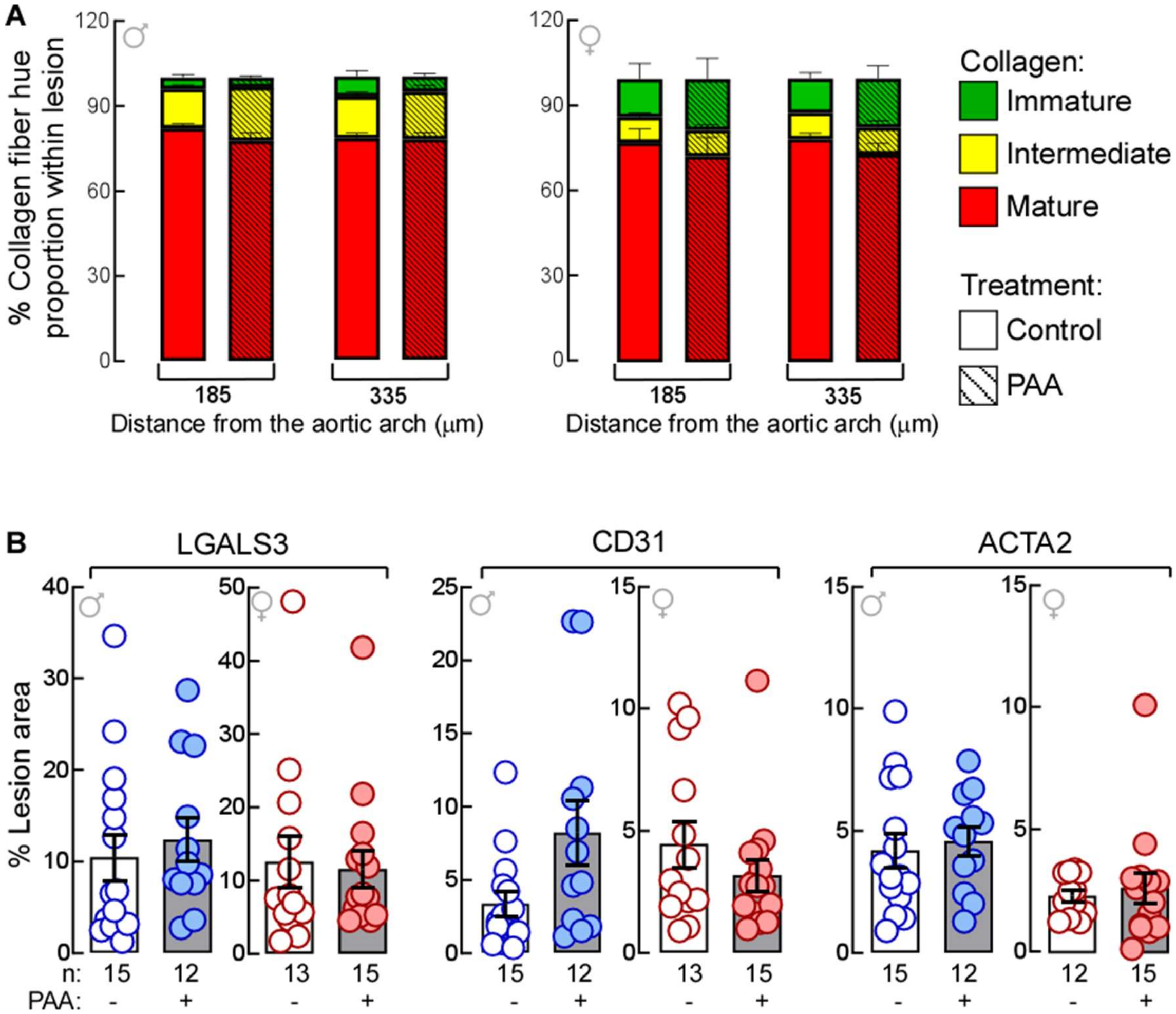

(**A**) Assessment of collagen fiber hue variations based on PicroSirius Red staining followed by polarized light microscopy. The ratio between immature (green), intermediate (yellow), and mature (red) collagen fibrils within the lesion of control and PAA-treated *Apoe*$^{-/-}$ mice; (**B**) LGALS3, CD31, and ACTA2 positive % of lesion area based on immunofluorescence staining within the control (open symbols) and PAA-treated (closed symbols) BCAs of male (blue) and female (red) mice. Data were analyzed by (**A**) mixed-model ANOVA, followed by Šídák's *post hoc* test and (**B**) Mann-Whitney test. Values = mean ± SEM; n = 11-14 as indicated. ACTA2 indicates smooth muscle α-actin 2; BCA, brachiocephalic artery; CD31, platelet endothelial cell adhesion molecule-1; LGALS3, galectin-3; and PAA, phenylacetic acid.

Figure S3 is related to **Figure 3**.

Figure S4. PAA alters gut microbial composition and changes circulating levels of 4-HPA, but it does not affect other metabolites associated with atherosclerosis.

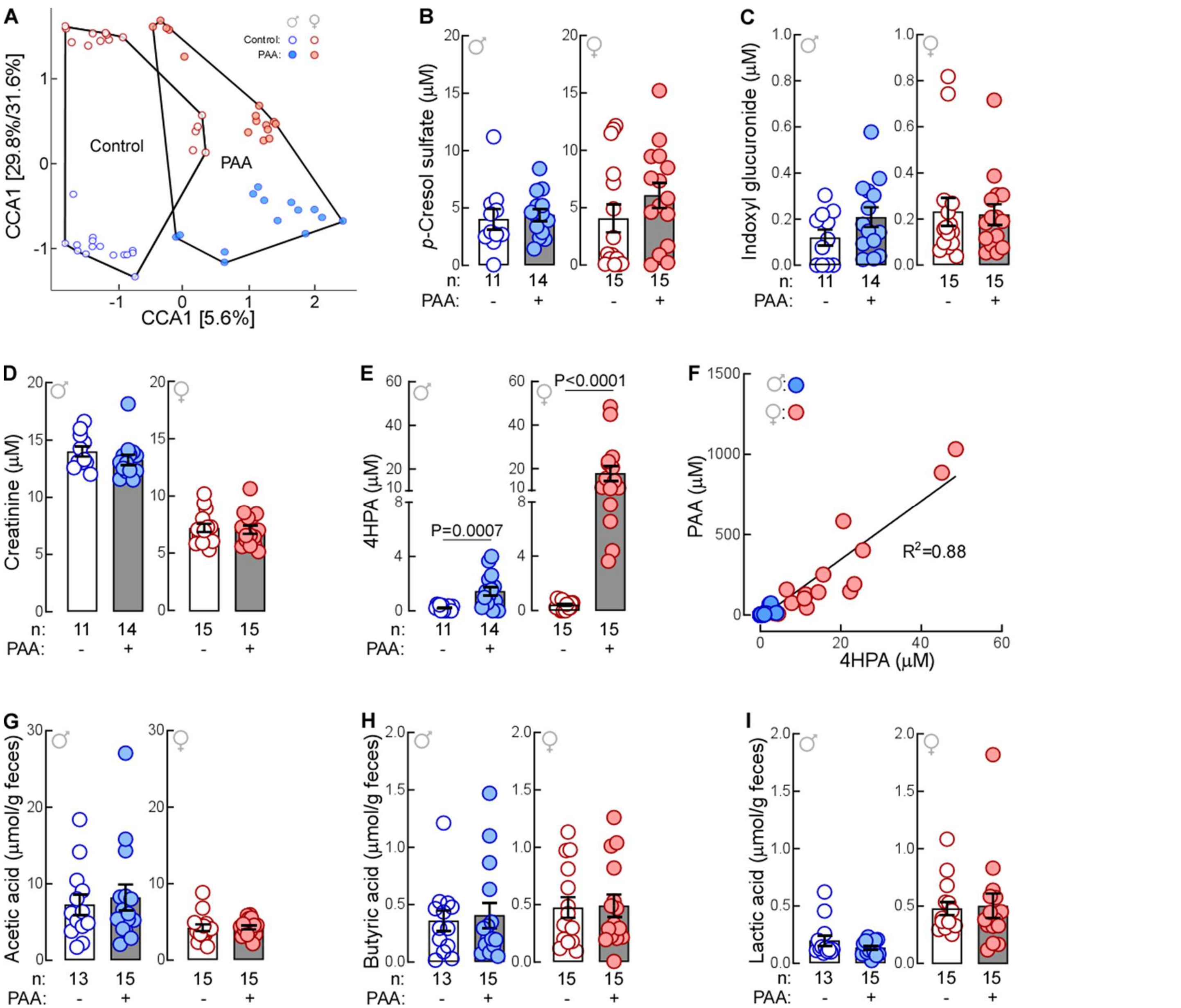

(**A**) CCA of overall beta diversity index among the indicated groups. Circulating levels of (**B**) *p*-cresol sulfate, (**C**) indoxyl glucuronide, (**D**) creatinine, and (**E**) 4HPA. (**F**) Correlation between circulating levels of PAA and 4HPA. Fecal levels of (**G**) acetic acid, (**H**) butyric acid, and (**I**) lactic acid in control (open symbols) and PAA-treated (closed symbols) male (blue) and female (red) *Apoe*$^{-/-}$ mice. Data were analyzed by Mann-Whitney test. Values = mean ± SEM; n = 11-15 as indicated. 4HPA indicates 4-hydroxyphenylacetic acid; CCA, canonical correlation analysis; and PAA, phenylacetic acid.

Figure S4 is related to **Figure 4**.

Figure S5. Dietary provision of PAA increases liver weight in female WT mice fed high-fat diet.

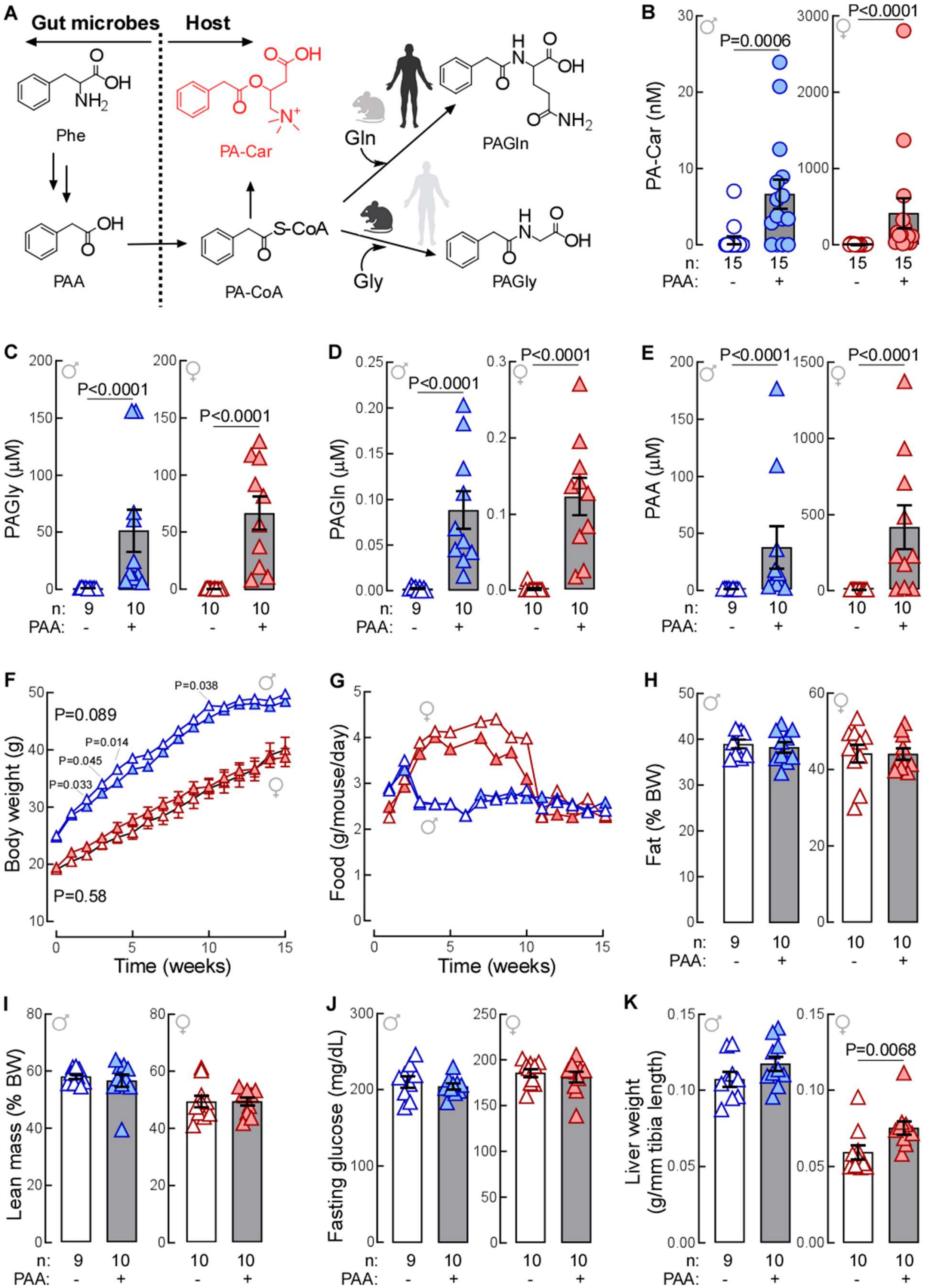

(**A**) Schematic representation of PA-Car synthesis from the PA-CoA intermediate. (**B**) Plasma levels of PA-Car in control (open symbols) and PAA-treated (closed symbols) male (blue) and female (red) *Apoe*$^{-/-}$ mice (circle) in the fed state. Circulating levels of (**C**) PAGly, (**D**) PAGln, and (**E**) PAA in control (open symbols) and PAA-treated (closed symbols) male (blue) and female (red) in WT (triangle) mice in the fed state. (**F**) Body weight and (**G**) food consumption over time in control (open symbols) and PAA-treated (closed symbols) male (blue) and female (red) WT (triangle) mice. Body composition established by EchoMRI represented as percent of body (**H**) fat or (**I**) lean mass in control (open symbols) and PAA-treated (closed symbols) of male (blue) and female (red) WT (triangle) mice. (**J**) Blood glucose levels measured in 4 h fasted animals, and (**K**) liver weights normalized to tibia length in control (open symbols) and PAA treated (closed symbols) of male (blue) and female (red) WT (triangle) mice. Mann-Whitney test (**B**-**E** and **H**-**K**) or mixed-model ANOVA with Tukey's *post hoc* test (**F**) was used for statistical analysis. Values = mean ± SEM; n = 9-15 as indicated. BW, indicates body weight; Gln, glutamine; Gly, glycine; PAA phenylacetic acid; PA-Car, phenylacetyl-carnitine; PA-CoA, phenylacetyl-coenzyme A; PAGln, phenylacetylglutamine; PAGly, phenylacetylglycine; Phe, phenylalanine; and WT, wild-type.

Figure S5 related to **Figure 5**.

Figure S6. Dietary provision of PAA does not affect liver expression of genes encoding enzymes involved in carnitine biosynthesis or most enzymes required for β-oxidation in either *Apoe*$^{-/-}$ or WT mice.

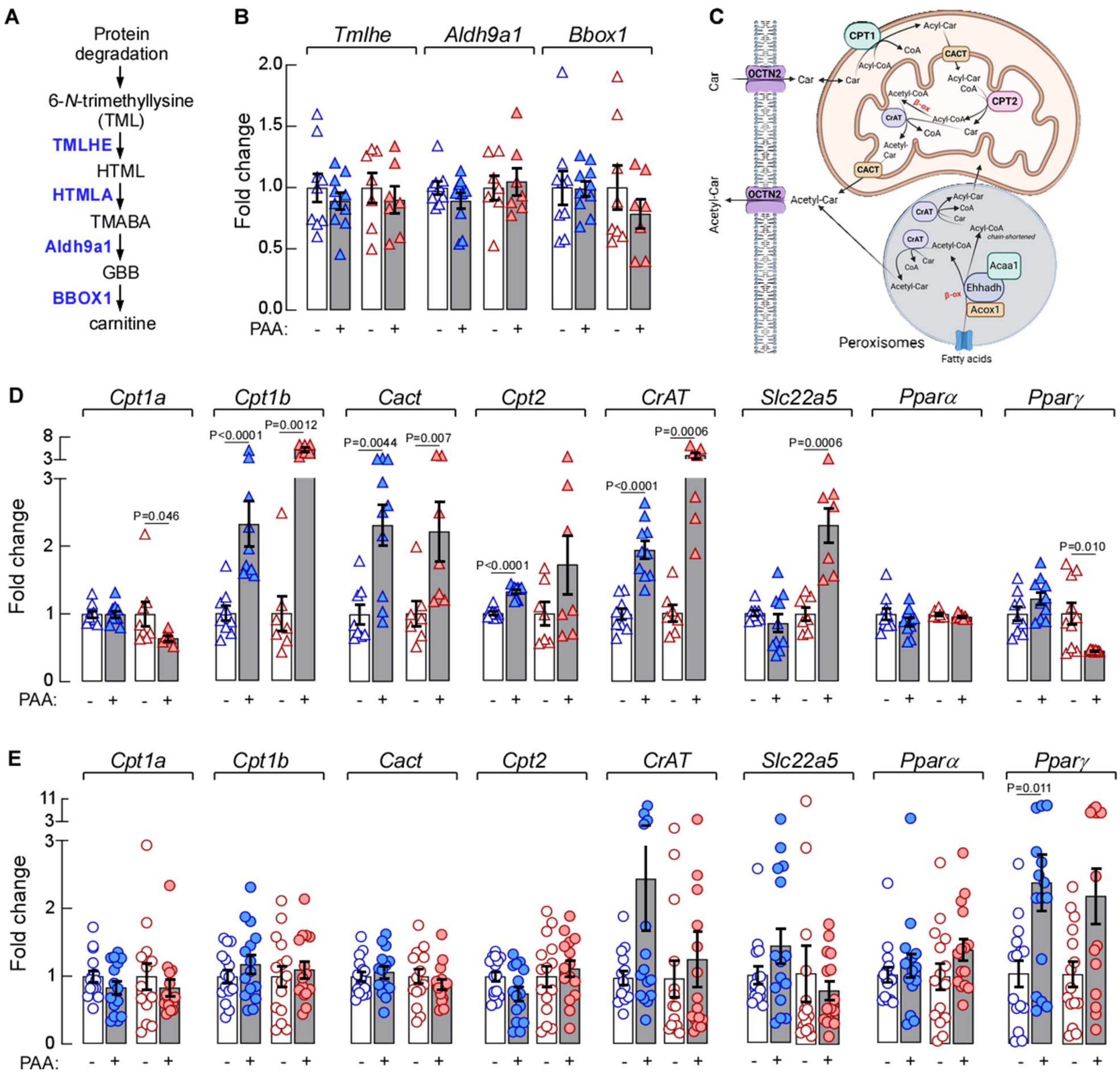

(**A**) Pathway of carnitine biosynthesis: TML is generated through protein degradation then hydroxylated by TMLHE into HTML. HTML is converted into TMABA by HTMLA and then oxidized to GBB by Aldh9a1. Carnitine is formed in the final step by BBOX1 hydrolysis of GBB. (**B**) Hepatic expression of indicated genes involved in carnitine biosynthesis in control (open symbols) and PAA treated (closed symbols) of male (blue) and female (red) WT (triangles) mice. (**C**) Mitochondrial and peroxisomal lipid metabolism with key

genes/enzymes depicted. (**D** and **E**) Hepatic expression of indicated genes in control (open symbols) and PAA treated (closed symbols) of male (blue) and female (red) WT (triangles) and *Apoe*$^{-/-}$ (circles) mice; Mann-Whitney test was used for statistical analysis. Values = mean ± SEM. Acaa1 indicates acetyl-coenzyme A acyltransferase 1; Acetyl-Car, acetyl-carnitine; Acox1, acyl- coenzyme A oxidase 1; Acyl-Car, acyl-carnitine; Acyl-CoA, acyl-coenzyme A; Aldh9a1, aldehyde dehydrogenase 9 family member A1; BBOX1, γ-butyrobetaine hydroxylase 1; β-ox, β-oxidation; CACT, carnitine-acylcarnitine translocase; Car, carnitine; CPT1, carnitine palmitoyltransferase 1; CPT2, carnitine palmitoyltransferase 2; CrAT, carnitine acetyltransferase; EHHADH, enoyl-coenzyme A hydratase/3-hydroxyacyl- coenzyme A dehydrogenase; GBB, γ-butyrobetaine; HTML, 3-hydroxy-6-*N*-trimethyllysine; HTMLA, 3-hydroxy-6-*N*-trimethyllysine aldolase; OCTN2, organic cation/carnitine transporter 2; PAA, phenylacetic acid; Pparα, peroxisome proliferator-activated receptor alpha; Pparaγ, Peroxisome proliferator-activated receptor gamma; SLC22a5, solute carrier family 22 Member 5 encoding (encodes OCTN2 protein); TMABA, 4-trimethylaminobutyraldehyde; TML, trimethyllysine; TMLHE, trimethyllysine dioxygenase; and WT, wild-type.

Figure S6 is related to **Figure 6**.
